# Pediatric health service utilization at tertiary hospitals in Denmark 2000–2018

**DOI:** 10.1038/s41598-024-63853-8

**Published:** 2024-06-06

**Authors:** Pi Vejsig Madsen, Andreas Jensen, Frank Eriksson, Lone Graff Stensballe

**Affiliations:** 1 Mary Elizabeth’s Hospital, Rigshospitalet, Copenhagen University Hospital, Copenhagen, Denmark; 2 Department of Paediatrics and Adolescent Medicine, Rigshospitalet, Copenhagen University Hospital, Copenhagen, Denmark; 3 Section of Biostatistics, Department of Public Health, University of Copenhagen, Copenhagen, Denmark; 4 Institute of Clinical Medicine, University of Copenhagen, Copenhagen, Denmark

**Keywords:** Health services, Paediatrics, Public health

## Abstract

Pediatric health service differs between and within countries. To prioritize limited resources, data-driven studies on pediatric tertiary hospital contacts are warranted. This population-based register study identified all contacts with four Danish tertiary hospitals 2000–2018 by 0–17-year-old patients. During 2000–2018, 2,496,001 individuals resided in Denmark while 0–17 years old, and the study described 829,562 inpatient and 3,932,744 outpatient contacts at tertiary hospitals by hospital, sex, age, diagnosis, department, and residence. Male patients accounted for more contacts overall (inpatient 55.51%, outpatient 52.40%) and more contacts with severe chronic disease (inpatient 56.24%, outpatient 54.41%). Median (interquartile range) patient age was 3.09 (0.26–9.96) and 8.48 (2.78–13.70) years for in- and outpatient contacts. Overall, 28.23% and 21.02% of in- and outpatient contacts included a diagnosis of a severe chronic disease, but the proportions differed across hospitals. A pattern of pediatric healthcare directed towards less severe diseases was observed: While the total number of outpatient visits at tertiary hospitals increased from 2000 to 2018, the proportion of these contacts which had a diagnosis of a severe chronic disease decreased. Future comparisons between hospitals regarding pediatric outcomes should consider potential differences in terms of uptake and diagnosis severity. Such findings may have implications for future pediatric organization, nationally and internationally.

## Introduction

Knowledge of the existing organization of tertiary healthcare for children and adolescents is vital to facilitate an understanding of organizational pros and cons, to carry out national and international comparisons to expand effective solutions, and to evaluate the impact of organizational changes. Indeed, it is necessary to consider preexisting differences and trends developing over time to properly isolate the effect of interventions on the healthcare structure.

Previously, collective initiatives described differences between pediatric healthcare in European countries at a national level^1,2^. This was done with a focus on the strengths and weaknesses arising due to the organization of healthcare in each country. To this end, descriptive statistics regarding health services in the form of, e.g., the number or rate of pediatric hospital beds and pediatric healthcare professionals were presented for many countries^3–9^, while others included information about pediatric admissions or outpatient visits^10–13^. However, providing exhaustive nationwide health statistics was not a main goal^1^. In particular, the quantification of pediatric practice in Denmark is scarce^14^. A Chinese study outlined pediatric capacities and hospital contacts at a national level using survey data, distinguishing between primary, secondary, and tertiary healthcare as well as geographical region^15^. Other studies also compared health services in different regions, often with a focus on a narrow age group. In this vein, studies have compared the neonatal hospital capacities and treatments for preterm births across all regions of the Nordic countries^16^ or across a selection of high-income countries^17^. Considerable differences in organization between regions and countries were found. In the Nordic countries, differences in the proportion of preterm births, patient characteristics, neonatal capacities, and admission rates were found, despite no significant difference in mortality or morbidity^16^, signaling clear organizational differences despite comparable populational needs. Thus, comprehensive numbers regarding tertiary pediatric hospital contacts at a national level are lacking. Nationwide administrative health registries, such as those available in Denmark, provide ample opportunities to produce such comprehensive statistics in the setting of a universal, tax funded, free-access, public healthcare system^18^. The literature search is described in the Supplementary Information.

Tertiary hospitals deliver highly specialized health services. As such, it is expected that a large proportion of pediatric services and resources at tertiary hospitals are allocated to the treatment of severe chronic diseases. A previous study found a higher rate of in- and outpatient contacts for children with a severe chronic disease compared to healthy children in a cohort of Danish children under five years of age^19^. The number of pediatric in- and outpatient contacts with tertiary hospitals in Denmark pertaining to a severe chronic disease has not been mapped out previously, nor has its development over the past years.

Thus, the objective of the present study was to describe the utilization of tertiary somatic health services in the form of admissions and outpatient visits among Danish children and adolescents aged 0–17 years old. This was accomplished with a longitudinal descriptive cross-sectional study based on the national cohort of Danish children and adolescents 2000–2018 using data from administrative registers. Changes over time within the four Danish tertiary hospitals were described. Additionally, the proportion of contacts pertaining to severe chronic diseases was determined. This enables future studies to properly consider comparability of health-related outcomes for pediatric patients at different periods of time and different tertiary hospitals in Denmark. Of immediate clinical relevance, such data-overviews facilitate informed leadership and prioritization of limited personnel and economic resources.

## Methods

### Data sources

Danish national registers were used to obtain data regarding demographics and contacts with the Danish public healthcare system for children and adolescents residing in Denmark. Individuals were linked between registers using their civil registration number (CPR-number), which allowed for unique identification of individuals^20^. Data was accessed in a pseudonymous form located in a secure server provided by Statistics Denmark^21^.

The Danish Medical Birth Register (MBR)^22^ and Civil Registration System (CRS) were used for demographic information including date of birth, sex assigned at birth, municipality, dates of migrations in or out of Denmark, and date of death. The Danish National Patient Registry (DNPR) was used for data regarding contacts with public hospitals^23^. This included data regarding date of admission and discharge, type of patient (inpatient, elective outpatient, or acute outpatient), the responsible hospital and department, as well as diagnoses in the form of International Classification of Diseases 10th Revision (ICD-10) codes^24^. During the years 2000–2018, DNPR was structured in records representing a group of contacts with a particular hospital for a patient. Visit dates associated with outpatient records were also available. An inpatient record constituted an admission.

### Ethical statement

The access to all underlying data was secured within the servers of Statistics Denmark who also approved the projection description of the study. All data were fully pseudonymized. The study was carried out in accordance with all applicable guidelines and regulations. According to Danish law, further ethics committee approval and informed consent are not required for register-based studies.

### Inclusion and exclusion criteria

A cross-sectional study design was employed. The study period was January 1, 2000, to December 31, 2018. It was chosen to have a long and recent period of observation prior to structural changes implemented to DNPR in 2019. For records in DNPR, and by extension outpatient visits and admissions (collectively referred to as “contacts”) at Danish public hospitals, the following inclusion criteria were employed:

- The patient was 0–17 years old (both included) at the start of the contact
- The patient resided in Denmark at the start of the record
- For outpatient visits: The visit occurred in the years 2000–2018
- For admissions: The patient was discharged in 2000–2018 or still admitted as of December 31, 2018
- The responsible hospital was tertiary (i.e., Rigshospitalet, Odense University Hospital, Aarhus University Hospital, or Aalborg University Hospital)

Along with this, the following exclusion criteria were applied:

i) The contact was an acute outpatient visit
ii) The contact only had psychiatric diagnoses
iii) The contact only had birth diagnoses

Note that the statistical units considered were *hospital contacts*, not individuals. The number of unique individuals represented in accordance with the above criteria is also presented in the results section.

The tertiary hospitals were defined as Rigshospitalet (part of Copenhagen University Hospital), Odense University Hospital, Aarhus University Hospital, and Aalborg University Hospital. Skejby University Hospital and Aarhus Hospital, two predecessors of Aarhus University Hospital, were included under the current name. Likewise, Aalborg Hospital, the predecessor of Aalborg University Hospital, was included under the current name (Supplementary Table S1).

Main, secondary, and additional diagnoses were considered. Referral diagnoses were excluded, as they are considered less reliable. Contacts containing only psychiatric diagnoses were excluded since the focus of the present study was somatic contacts. Generally, psychiatric diagnoses were not considered in summaries of the most frequent diagnoses. In order to focus on contacts indicating disease or need of care, admissions with *only* diagnoses pertaining to birth were excluded. In Denmark the vast majority of individuals are born at hospitals—a contact which does not per se signal disease nor need of further care in hospital. Thus, we would like to present the activity in pediatrics besides births. However, note that not all births (i.e., contacts with main diagnosis ICD-10 Z38) were excluded, as contacts pertaining to birth followed by additional care for the child could be of interest. See the Supplementary Information page 6 for the definition of birth diagnoses used. Finally, acute outpatient visits were excluded, since maintenance of, e.g., emergency rooms is not a tertiary task.

To contextualize the number of admissions, outpatient visits, and unique patients, the background population was defined as individuals residing in Denmark at some point between January 1, 2000, and December 31, 2018, while being 0–17 years old. The person-time spent in Denmark while 0–17 years old was determined in total and by calendar year.

To be able to inspect the potential impact of, e.g., centralization and task shifting, the number of contacts per year at secondary hospitals (i.e., public somatic hospitals in Denmark excluding the four tertiary hospitals) was also determined. This was done employing the inclusion criteria (a) through (d), with (e) replaced by secondary hospitals as defined in the Supplementary Information pages 3–4, along with the exclusion criteria (i) through (iii).

### Outcomes and categorization

The version of CRS used was a year-end intersection of the Danish population. To account for early death, hospital contacts from individuals appearing in MBR 2000–2018 or in CRS 2000–2018 were considered for inclusion. People born in Denmark, i.e., individuals in MBR 2000–2018, were assumed to reside in Denmark until emigration or death. Sex and date of birth were based on MBR if possible, otherwise on CRS. See the Supplementary Information for details regarding data management and categorization.

The number of admissions and outpatient visits at tertiary hospitals were co-primary outcomes. The outcomes were described by admission duration, patient sex, patient age, presence of a diagnosis of a severe chronic disease, diagnoses, hospital department, and the patients’ place of residence. The number of unique patients with admissions or outpatient visits was also described.

### Admissions and outpatient visits

Patients were categorized as inpatients or elective outpatients (referred to simply as outpatients). The admission and discharge dates for inpatients were amended such that no patient was hospitalized in two or more departments simultaneously. Admissions within one hospital with admission the same day as the previous discharge date were counted as one admission. The number of admission days was also considered. An admission with same-day discharge was counted as lasting one day. Thus, all admissions had a duration of at least one day.

Some outpatient records had no visit dates available. Most of these records were of short duration, making it plausible that only one visit occurred, and the start of the record was assumed to be the sole visit date. The handling of records and visit dates is explained on page 5 of the Supplementary Information.

### Severe chronic disease

Severe chronic disease in children was defined using ICD-10 codes. See Supplementary Table S3 for the definition, which is based on potentially life-threatening diagnoses. The same definition has been used previously, and severe chronic disease defined in this manner was indeed found to be strongly associated with mortality for children under five years old in Denmark^25^. A contact was defined to pertain to a severe chronic disease if the contact included at least one diagnosis of a severe chronic disease.

### Residence

The five Danish Regions (Capital Region of Denmark, Region Zealand, Region of Southern Denmark, Central Denmark Region, and North Denmark Region) were established in 2007. They are responsible for administering healthcare, including general practitioners, other specialists in private practice, as well as hospitals^18^. Despite the introduction of the Regions occurring during the study period, they were used to group the patients’ residence, due to their manageable number and geographical relevance. The four tertiary hospitals are in four different Regions. To provide context for the relative sizes of the child-populations of the Regions, the approximate percentage of person-time spent in each Region for the background population was calculated by assuming that the region of residence at the end of the previous year extended to the following year. For instance, an individual’s residence as of December 31, 2000, carried forward to 2001, and their person-time was attributed to said Region. The Region of individuals not present in CRS the previous year (e.g., due to birth or immigration) was considered missing, and thus not included. The calendar year 2000 was not included in this calculation due to lack of data from 1999.

Incidence rates were calculated relative to the person-time spent in Denmark while 0–17 years old. While any individual satisfying these two criteria could in principle have a contact with any one of the four tertiary hospitals, each hospital has an uptake population depending on mostly geography but also diagnoses (due to centralization of certain treatments). Thus, the uptake population for each tertiary hospital is not entirely straightforward to define.

### The Danish Healthcare System

In Denmark, basic healthcare is taxpayer-funded and provided free of charge. Danish healthcare is provided by three sectors: primary healthcare includes family physicians, other private specialist physicians, and nursing homes; secondary healthcare consists of hospitals; and within the hospital system, four hospitals are denoted as tertiary based on their level of specialization. The four tertiary hospitals are Aalborg University Hospital, Aarhus University Hospital, Odense University Hospital, and Copenhagen University Hospital, which mainly consists of ‘Rigshospitalet’. Generally, hospital patients in need of tertiary care are referred from a secondary hospital to the regional tertiary hospital, which is Aalborg University Hospital for the North Denmark Region, Aarhus University Hospital for the Central Denmark Region, Odense University Hospital for the Region of Southern Denmark, and Copenhagen University Hospital for Region Zealand and the Capital Region of Denmark. Pediatric oncology is an example of specialized treatment centralized to the tertiary hospitals. Further, since Denmark is a small country with a population of 5.8 million inhabitants, some areas within pediatrics are centralized to one tertiary hospital only, for example all patients with cleft palate and congenital heart disease are treated primarily at Copenhagen University Hospital.

### Software

Data management and visualization was done using R version 4.3.1^26^ and the Tidyverse-libraries^27^.

## Results

During the study period 2000–2018, 2,496,001 individuals 0–17 years old resided in Denmark (22,627,930 person-years). The number of individuals residing in Denmark while 0–17 years old per year remained stable throughout 2000–2018. The maximum number of individuals was reached in 2008 (Supplementary Fig. S2 and Supplementary Table S4). The same development was observed for person-time (Supplementary Fig. S3 and Supplementary Table S5). Approximately 29.3% of the person-time was spent in the Capital Region of Denmark, 14.8% in Region Zealand, 22.0% in the Region of Southern Denmark, 23.5% in the Central Denmark Region, and 10.4% in the North Denmark Region.

**Figure 1:**
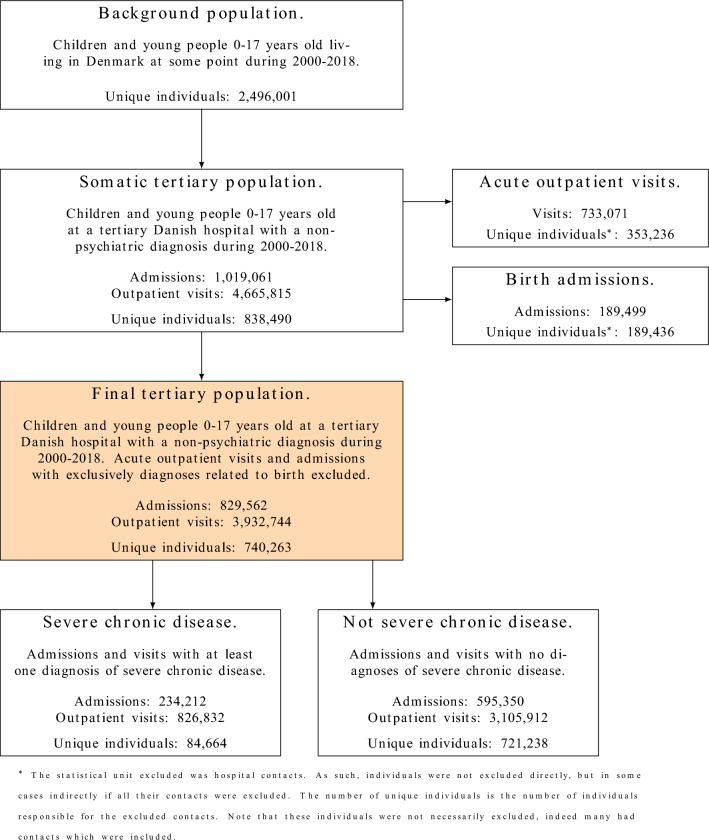
The number of included and excluded admissions and outpatient visits. The orange box contains the main population, which is further subdivided into contacts pertaining to a severe chronic disease and contacts not pertaining to a severe chronic disease.

The selected tertiary contacts consisted of 829,562 admissions and 3,932,744 outpatient visits from 740,263 unique individuals. A flowchart illustrating the number of included and excluded hospital contacts is found in Fig. 1. Information regarding excluded patients, along with brief description of acute outpatient visits and admissions with only birth diagnoses, is in the Supplementary Information. Table 1 outlines characteristics of the tertiary admissions and outpatient visits.

**Table 1:** Admissions and outpatient visits 2000–2018 at the four tertiary hospitals in Denmark. Male patients, severe chronic disease, pediatric department, and region are listed in number of contacts with percentages relative to the hospital. Age is the median (interquartile range) age in years at start of contact.

	Rigshospitalet	Odense University Hospital	Aarhus University Hospital	Aalborg University Hospital	Overall
**Admissions**	252,151	214,529	220,360	142,522	829,562
Unique patients	100,705	104,731	101,106	78,379	371,980
Days in hospital With severe chronic disease	1,164,657 565,807 (48.58%)	843,493 233,999 (27.74%)	889,904 314,411 (35.33%)	585,586 106,597 (18.20%)	3,483,640 1,220,814 (35.04%)
Male patients	141,477 (56.12%)	119,319 (55.62%)	122,919 (55.78%)	76,839 (53.91%)	460,554 (55.51%)
Age	3.81 (0.54–10.33)	2.28 (0.17–8.93)	3.78 (0.49–10.65)	2.05 (0.00–9.31)	3.09 (0.26–9.96)
Severe chronic disease	102,294 (40.57%)	41,071 (19.14%)	70,310 (31.91%)	20,537 (14.41%)	234,212 (28.23%)
Pediatric department	163,481 (64.83%)	118,872 (55.41%)	133,089 (60.40%)	74,748 (52.45%)	490,190 (59.09%)
Patients from hospital’s region	189,839 (75.29%)	198,949 (92.74%)	184,109 (83.55%)	139,003 (97.53%)	711,900 (85.82%)
**Outpatient visits**	1,075,168	1,144,540	1,057,005	656,031	3,932,744
Unique patients	179,700	176,290	203,176	134,357	665,273
Male patients	576,626 (53.63%)	597,785 (52.23%)	553,447 (52.36%)	333,038 (50.77%)	2,060,896 (52.40%)
Age	7.71 (2.46–13.11)	8.97 (3.07–14.03)	8.19 (2.45–13.48)	9.42 (3.45–14.36)	8.48 (2.78–13.70)
Severe chronic disease	366,045 (34.05%)	189,697 (16.57%)	197,559 (18.69%)	73,531 (11.21%)	826,832 (21.02%)
Pediatric department	541,196 (50.34%)	387,366 (33.84%)	376,183 (35.59%)	193,000 (29.42%)	1,497,745 (38.08%)
Patients from hospital’s region	840,213 (78.15%)	1,093,154 (95.51%)	926,175 (87.62%)	645,327 (98.37%)	3,504,869 (89.12%)

**Figure 2:**
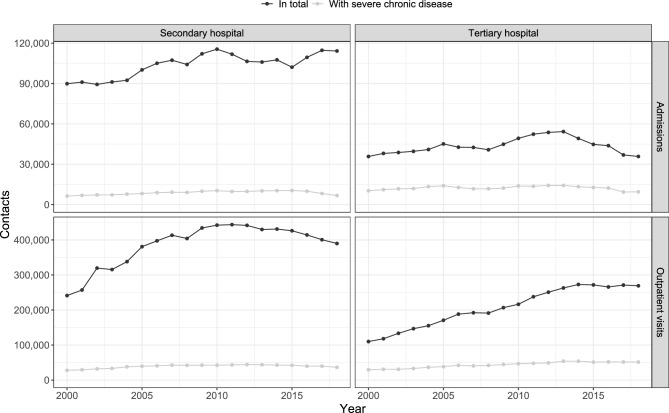
Admissions and outpatient visits at secondary and tertiary hospitals. Contacts starting prior to 2000 are not shown. See Supplementary Fig. S8 for the corresponding incidence rates.

In addition, 1,971,559 admissions and 7,322,829 outpatient visits at secondary hospitals were identified (compromising 1,582,945 patients), see the Supplementary Information page 14 for details regarding the development over time. Details regarding patient characteristics and diagnoses of contacts at the secondary hospitals are provided in the Supplementary Information on page 22 (Supplementary Tables S9–S11). Figure 2 shows the number of tertiary and secondary hospital contacts over time (see Supplementary Tables S7, S8 for the corresponding numbers). Admissions at secondary hospitals increased from 2000 to 2018 (by 24,309 admissions, 27.0% increase relative to the year 2000), however, the trend was not uninterrupted throughout the period. Although the number of admissions at tertiary hospitals in 2000 and 2018 were essentially equal, the number of admissions did not remain constant throughout 2000–2018. In particular, the number of admissions at tertiary hospitals peaked in 2013, followed by a decrease back to the initial level. From 2000 to 2010, both tertiary and secondary hospitals saw an increase in outpatient visits per year. Afterwards, from 2010 to 2018, the number of outpatient visits at tertiary hospitals continued to increase (52,860 more visits in 2018 compared to 2010) while the number decreased correspondingly at secondary hospitals (by 52,362 visits).

### Admissions at tertiary hospitals

All four hospitals had most male patients. Rigshospitalet had the highest percentage of male patients (56.1%), while Aalborg University Hospital had the lowest (53.9%), see Table 1. The proportion of male inpatients was most pronounced among one-year-old patients (58.9%). Female patients were more frequent for adolescents 14–17 years old. For ages 14–16 years the margin was slight, around 50.7% female, while for age 17 years the percentage was 52.0%. Supplementary Figure S13 shows more details on sex and age. Supplementary Table S12 contains information on sex and severe chronic diseases.

Infants had by far the highest frequency of admission, accounting for 278,564 admissions (33.6%), succeeded by age one year with 82,317 admissions (9.9%). The number of admissions decreased from ages zero to seven and then remained stable (Supplementary Fig. S14). The same trend was seen within each of the four hospitals (Supplementary Fig. S15).

**Figure 3:**
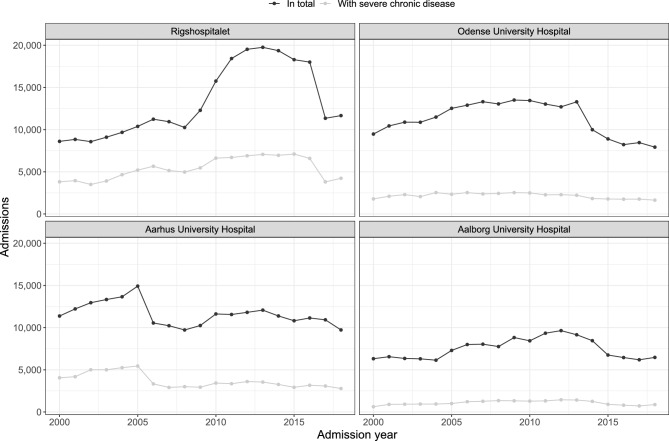
Admissions at the four tertiary hospitals by admission year. A limited number of included admissions started in 1999 and are not shown here. See Supplementary Fig. S16 for the corresponding incidence rates.

The development in the number of admissions over time along with the number of these connected to a severe chronic disease is seen in Fig. 3 (also see Supplementary Tables S7, S13, and S14). Considering incidence rates instead of absolute number of admissions changed little (Supplementary Fig. S16), since the underlying population of children and adolescents residing in Denmark varied minimally during 2000–2018. In total, 40.6% of admissions at Rigshospitalet included a diagnosis of a severe chronic disease, compared to 19.1% at Odense University Hospital, 31.9% at Aarhus University Hospital, and 14.4% at Aalborg University Hospital (Table 1).

The most common main diagnosis for admissions was birth (ICD-10: Z38), with 130,002 admissions (15.7%). Recall that while admissions with exclusively birth diagnoses were excluded, many birth related admission were still included due to the presence of other diagnoses of potential interest. Table 2 contains the ten most common non-birth main diagnoses for admissions. Overall, the most common non-birth diagnosis was medical observation (ICD-10: Z03). An exploration of secondary diagnoses for admissions with main diagnosis Z03 (medical observation) and Z76 (contact with the healthcare system for other reasons than disease, i.e., routine health check), another common admission diagnosis, did not reveal much additional information regarding the underlying reasons for admission (Supplementary Table S16). The most common main diagnosis of a severe chronic disease was lymphoid leukemia (3.9% of all admissions, 13.8% of admissions with a severe chronic disease). It was even the overall most common non-birth diagnosis at Rigshospitalet. Lymphoid leukemia was also the most common diagnosis of a severe chronic disease at the other hospitals. In total, 1217 unique individuals were admitted with lymphoid leukemia (1508 individuals had an in- or outpatient contact with lymphoid leukemia). See Supplementary Table S15 for more details on admissions with a diagnosis of a severe chronic disease.

**Table 2:** The ten most common non-birth main diagnoses (3-digit ICD-10 code) for admissions at the four tertiary hospitals. Percentages were calculated relative to the total number of admissions, which might have more than one main diagnosis due to consolidation of adjacent records.

	ICD-10		n (%)
For the hospitals combined			
1	Z03	Medical observation for suspected diseases/conditions, ruled out	54,614 (6.58)
2	C91	Lymphoid leukemia	32,232 (3.89)
3	P07	Disorders related to short gestation and low birth weight	20,518 (2.47)
4	J45	Asthma	16,582 (2.00)
5	R10	Abdominal and pelvic pain	14,314 (1.73)
6	R56	Convulsions, not elsewhere classified	13,667 (1.65)
7	B34	Viral infection of unspecified site	11,429 (1.38)
8	Z76	Encountering health services in other circumstances	11,343 (1.37)
9	M08	Juvenile arthritis	9841 (1.19)
10	J18	Pneumonia, organism unspecified	9757 (1.18)
For Rigshospitalet			
1	C91	Lymphoid leukemia	14,589 (5.79)
2	Z03	Medical observation for suspected diseases/conditions, ruled out	13,695 (5.43)
3	M08	Juvenile arthritis	5804 (2.30)
4	P07	Disorders related to short gestation and low birth weight	5535 (2.20)
5	Z76	Encountering health services in other circumstances	4016 (1.59)
6	Q37	Cleft palate with cleft lip	3989 (1.58)
7	Z94	Transplanted organ and tissue status	3562 (1.41)
8	J96	Respiratory failure, not elsewhere classified	3357 (1.33)
9	C71	Malignant neoplasm of brain	3243 (1.29)
10	Q21	Congenital malformations of cardiac septa	3061 (1.21)
For Odense University Hospital			
1	Z03	Medical observation for suspected diseases/conditions, ruled out	14,003 (6.53)
2	J45	Asthma	8271 (3.86)
3	P07	Disorders related to short gestation and low birth weight	5279 (2.46)
4	R10	Abdominal and pelvic pain	4775 (2.23)
5	R56	Convulsions, not elsewhere classified	4740 (2.21)
6	B34	Viral infection of unspecified site	4503 (2.10)
7	J18	Pneumonia, organism unspecified	3884 (1.81)
8	C91	Lymphoid leukemia	3706 (1.73)
9	K59	Other functional intestinal disorders	3608 (1.68)
10	J05	Acute obstructive laryngitis and epiglottitis	3426 (1.60)
For Aarhus University Hospital			
1	Z03	Medical observation for suspected diseases/conditions, ruled out	13,170 (5.98)
2	C91	Lymphoid leukemia	10,118 (4.59)
3	P07	Disorders related to short gestation and low birth weight	5916 (2.68)
4	J45	Asthma	3913 (1.78)
5	R10	Abdominal and pelvic pain	3896 (1.77)
6	Q62	Congenital obstructive defects of renal pelvis and congenital malformations of ureter	3817 (1.73)
7	R56	Convulsions, not elsewhere classified	3266 (1.48)
8	Q54	Hypospadias	3086 (1.40)
9	M08	Juvenile arthritis	2690 (1.22)
10	R50	Fever of other and unknown origin	2667 (1.21)
For Aalborg University Hospital			
1	Z03	Medical observation for suspected diseases/conditions, ruled out	13,746 (9.64)
2	Z76	Encountering health services in other circumstances	5994 (4.21)
3	C91	Lymphoid leukemia	3819 (2.68)
4	P07	Disorders related to short gestation and low birth weight	3788 (2.66)
5	J35	Chronic diseases of tonsils and adenoids	3600 (2.53)
6	R56	Convulsions, not elsewhere classified	3579 (2.51)
7	R10	Abdominal and pelvic pain	3541 (2.48)
8	J45	Asthma	3377 (2.37)
9	B34	Viral infection of unspecified site	2685 (1.88)
10	J20	Acute bronchitis	2639 (1.85)

In total, 3,483,640 days were spent in tertiary hospitals. For many admissions (261,032, 31.5%) the patient was admitted and discharged the same day. The number of days spent in hospital per year at Odense University Hospital and Aalborg University Hospital mirror the shape of the number of new admissions closely (compare Supplementary Fig. S17 to Fig. 3). At Rigshospitalet and Aarhus University Hospital it also mirrors the number of admissions, but with less drastic increases and decreases. Compared to the number of admissions, more days spent admitted pertained to a severe chronic disease (Table 1). Indeed, admissions with a diagnosis of a severe chronic disease generally lasted longer (Supplementary Fig. S18). Admissions starting in 2000 lasted a shorter time than admissions starting in 2010 and 2018 (Supplementary Fig. S19). The number of unique inpatients per year followed a similar trajectory as the number of admissions (Supplementary Fig. S20).

The inclusion and exclusion criteria required the patient to be 0–17 years old. However, the hospital departments did not need to be pediatric (see Supplementary Table S2 for the definition). Rigshospitalet had the highest percentage of admissions at pediatric departments (64.8%); Aalborg University Hospital the lowest (52.4%), see Table 1. The percentage of admissions at pediatric departments was highly age-dependent, with a larger proportion among younger patients (Supplementary Fig. S21). No substantial differences were seen over time (Supplementary Fig. S22). Admissions at non-pediatric departments occurred predominately at departments specializing in gynecology and obstetrics (97,796 admissions, 33.0% of admissions not at a pediatric department), orthopedic surgery (66,026 admissions, 22.3%), otorhinolaryngology (35,432 admissions, 12.0%). The many admissions to gynecology and obstetrics departments were mainly due to admissions pertaining to birth.

Finally, the residence of the patient was considered (Supplementary Table S17). At each of the four hospitals, most admissions were of patients living in the Region of the hospital (Table 1). These percentages were generally unchanged by the introduction of the Regions in 2007, excluding a modest increase at Rigshospitalet (Supplementary Fig. S23).

### Outpatient visits at tertiary hospitals

For outpatients, 3,932,744 visits from 665,273 unique patients satisfied the inclusion and exclusion criteria. Of these patients, 296,990 (44.6%) also experienced at least one admission.

Male patients had most outpatient visits, 2,060,896 (52.4%). For younger patients, the percentage was even larger, while most visits from patients 14–17 years old were by female patients (Supplementary Fig. S24). Male patients made up an even larger proportion of outpatient visits with a severe chronic disease (0.9–2.5 percentage point higher for the hospitals), see Supplementary Table S18.

Infancy was the most common age with 583,393 (14.8%) outpatient visits. The second and third most common ages were one year (247,080, 6.3%) and 17 years (242,188, 6.2%). See Supplementary Figs. S25 and S26 for more details on age.

**Figure 4:**
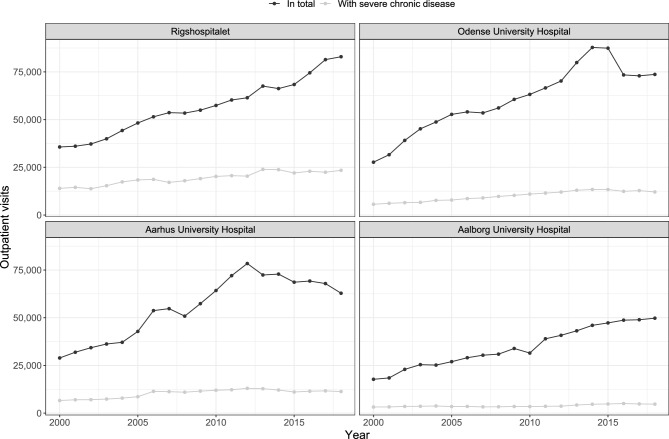
Outpatient visits at the four tertiary hospitals by year. A limited number of the included records did not have available visit dates and started prior to 2000, these are not shown here. See Supplementary Fig. S27 for the corresponding incidence rates.

Figure 4 shows the number of included outpatient visits at the four hospitals, along with the number of visits with a diagnosis of a severe chronic disease (see also Supplementary Tables S8, S19, and S20). From 2000 to 2018, the number of outpatient visits increased at all four hospitals (as did the number of unique outpatients, see Supplementary Fig. S28). The tertiary hospitals combined saw a 144.7% increase in outpatient visits from 2000 to 2018. The number of outpatient visits with a diagnosis of a severe chronic disease did not increase as much: by 75.0% in the same period. Replacing absolute number of outpatient visits by incidence rates makes little difference, see Supplementary Fig. S27. In total, 34.0% of visits at Rigshospitalet, 16.6% at Odense University Hospital, 18.7% at Aarhus University Hospital, and 11.2% at Aalborg University Hospital had a diagnosis of a severe chronic disease (Table 1).

The most common main diagnoses for outpatient visits are shown in Table 3. Overall, the four most common main diagnoses were observation and examination diagnoses (ICD-10: Z01, Z03, Z13, and Z09). These four diagnoses accounted for 23.5% of outpatient visits. Secondary diagnoses for outpatient visits with one of these four main diagnoses were considered (Supplementary Table S22). Not much information was gained as most of these outpatient visits did not include any secondary diagnoses. The most common diagnoses of severe chronic diseases for outpatient visits are presented in Supplementary Table S21. In total, lymphoid leukemia was the most frequent main diagnosis of a severe chronic disease for outpatient visits (1.5% of all visits, 6.9% of visits with a severe chronic disease). It was also the most common diagnosis of a severe chronic disease for outpatient visits at Rigshospitalet (type 1 diabetes at the other three hospitals).

**Table 3:** The ten most common main diagnoses (3-digit ICD-10 code) for outpatient visits at the four tertiary hospitals.

	ICD-10		n (%)
For the hospitals combined			
1	Z01	Other special examinations and investigations of persons without complaint	360,682 (9.17)
2	Z03	Medical observation for suspected diseases/conditions, ruled out	282,374 (7.18)
3	Z13	Special screening examinations for other diseases and disorders	158,735 (4.04)
4	Z09	Follow-up examination after treatment for conditions other than malignant neoplasms	120,596 (3.07)
5	J45	Asthma	102,911 (2.62)
6	M08	Juvenile arthritis	73,923 (1.88)
7	C91	Lymphoid leukemia	57,416 (1.46)
8	K07	Dentofacial anomalies	57,294 (1.46)
9	E10	Type 1 diabetes mellitus	47,976 (1.22)
10	H50	Other strabismus	45,853 (1.17)
For Rigshospitalet			
1	Z03	Medical observation for suspected diseases/conditions, ruled out	59,046 (5.49)
2	Z13	Special screening examinations for other diseases and disorders	35,484 (3.30)
3	C91	Lymphoid leukemia	33,100 (3.08)
4	M08	Juvenile arthritis	32,271 (3.00)
5	Z00	General examinations and investigations of persons without complaint	29,644 (2.76)
6	E84	Cystic fibrosis	24,619 (2.29)
7	E34	Other endocrine disorders	23,005 (2.14)
8	Z08	Follow-up examination after treatment for malignant neoplasms	20,937 (1.95)
9	Z94	Transplanted organ and tissue status	16,770 (1.56)
10	Q53	Undescended testicle	16,147 (1.50)
For Odense University Hospital			
1	Z01	Other special examinations and investigations of persons without complaint	143,988 (12.58)
2	Z03	Medical observation for suspected diseases/conditions, ruled out	75,955 (6.64)
3	J45	Asthma	47,766 (4.17)
4	Z13	Special screening examinations for other diseases and disorders	29,469 (2.57)
5	Z50	Care involving use of rehabilitation procedures	29,431 (2.57)
6	E10	Type 1 diabetes mellitus	20,241 (1.77)
7	L20	Atopic dermatitis	18,930 (1.65)
8	Z09	Follow-up examination after treatment for conditions other than malignant neoplasms	18,296 (1.60)
9	S52	Fracture of forearm	15,427 (1.35)
10	Z76	Encountering health services in other circumstances	14,113 (1.23)
For Aarhus University Hospital			
1	Z01	Other special examinations and investigations of persons without complaint	104,314 (9.87)
2	Z03	Medical observation for suspected diseases/conditions, ruled out	68,663 (6.50)
3	Z13	Special screening examinations for other diseases and disorders	61,871 (5.85)
4	Z09	Follow-up examination after treatment for conditions other than malignant neoplasms	30,705 (2.90)
5	M08	Juvenile arthritis	29,926 (2.83)
6	J45	Asthma	24,535 (2.32)
7	R32	Unspecified urinary incontinence	21,801 (2.06)
8	E10	Type 1 diabetes mellitus	16,542 (1.56)
9	E84	Cystic fibrosis	16,479 (1.56)
10	H90	Conductive and sensorineural hearing loss	15,787 (1.49)
For Aalborg University Hospital			
1	Z01	Other special examinations and investigations of persons without complaint	102,669 (15.65)
2	Z03	Medical observation for suspected diseases/conditions, ruled out	78,710 (12.00)
3	Z09	Follow-up examination after treatment for conditions other than malignant neoplasms	70,682 (10.77)
4	Z13	Special screening examinations for other diseases and disorders	31,911 (4.86)
5	K07	Dentofacial anomalies	19,927 (3.04)
6	J45	Asthma	19,425 (2.96)
7	H50	Other strabismus	13,044 (1.99)
8	E10	Type 1 diabetes mellitus	11,032 (1.68)
9	H90	Conductive and sensorineural hearing loss	8608 (1.31)
10	H52	Disorders of refraction and accommodation	7745 (1.18)

Of the 3,932,744 outpatient visits, 1,497,745 (38.1%) occurred at pediatric departments (Supplementary Table S2). Rigshospitalet had the highest proportion of visits at pediatric departments (50.3%); Aalborg University Hospital the lowest proportion (29.4%), see Table 1. The proportion was highly age-dependent with more outpatient visits at pediatric departments for younger patients (Supplementary Fig. S29). For the development over time see Supplementary Fig. S30. The outpatient visits at non-pediatric departments mostly occurred at departments specializing in orthopedic surgery (491,697 visits, 20.2% of visits not at a pediatric department), radiology (301,496 visits, 12.4%), and otorhinolaryngology (285,822 visits, 11.7%).

At each of the four hospitals, most outpatient visits were made by patients residing in the Region of the hospital (Table 1 and Supplementary Table S23). These percentages remained somewhat constant over time (Supplementary Fig. S31).

## Discussion

This nationwide register-based cross-sectional study included 2,496,001 Danish residents aged 0–17 years old during the period 2000–2018 and identified 829,562 admissions and 3,932,744 outpatient visits at four tertiary hospitals. These contacts were described in terms of sex, age, and diagnoses. Most patients were male, and infants accounted for a large proportion of contacts. Observational diagnoses were frequent. Of the severe chronic diseases, lymphoid leukemia was a prominent driver of tertiary health service utilization. During 2000–2018 the number of admissions first increased before decreasing; maybe explained by treatment needs increasingly being met by outpatient visits or home management. The number of outpatient visits increased substantially. Many differences between tertiary hospitals were observed, especially in the proportion of contacts with a severe chronic disease.

The number of outpatient visits was almost five-fold higher than the number of admissions. Almost all admitted patients also had an outpatient visit, indicating that outpatient visits were preferred when possible. Inpatients were younger than outpatients, and teenagers were more common among outpatients, which could be due to disease patterns changing with age. Male predominance was observed among inpatient contacts. This could be explained by male predominance among admissions in the younger age groups, which accounted for a large proportion of admissions. Most admissions occurred at pediatric departments, however, admission outside pediatric departments was frequent and increasing with patient age. The burden of severe chronic diseases was larger among admissions. Observational diagnoses dominated outpatient visits to a greater degree. This, along with the higher prevalence of severe chronic diseases for admissions, underlined the increased severity of disease leading to admissions. Probably reflecting the societal prioritization of timely treatment of malignant disease^28^, lymphoid leukemia was a frequent diagnosis for both in- and outpatient contacts. Despite only 1508 unique patients being seen with the diagnosis, a total of 116,874 contacts with lymphoid leukemia as a diagnosis were observed. Since cancer treatment has received much focus and timely protocolled cancer treatment became a legal requirement in Denmark in 2008, such findings reflect how societal priorities influenced health service utilization^29,30^. Type 1 diabetes was prominent for outpatient visits with a severe chronic disease but did not make the overall top-10 for admissions with a severe chronic disease. Such observations are important in our understanding of healthcare organization and utilization. Obviously, the organization reflected the population’s needs, but also political and societal priorities.

The number of admissions per year during 2000–2018 was seen to first increase before decreasing, the timing and degree of change differed between hospitals. Comparing 2000 to 2018, the number of admissions decreased at Odense University Hospital and Aarhus University Hospital, increased at Rigshospitalet, and was unchanged at Aalborg University Hospital. Contrast this with the number of outpatient visits, which increased dramatically over the study period at all four tertiary hospitals. These patterns may reflect a higher degree of specialization at Rigshospitalet, and a general tendency towards more outpatient contacts and home healthcare services, with fewer and shorter admissions, in line with the healthcare system’s increased focus on keeping patients in their normal surroundings whenever possible. Task shifting between secondary and tertiary hospitals provides another possible explanation for the increase in outpatient visits at tertiary hospitals. Indeed, the number of outpatient visits increased at both secondary and tertiary hospitals from 2000 to 2010 and continued to increase from 2010 to 2018 at tertiary hospitals, while it decreased correspondingly at the secondary hospitals. Notably, the number of contacts at tertiary hospitals with a diagnosis of a severe chronic disease increased at a lower rate compared to the overall number of contacts during 2000–2018. Thus, pediatric care at tertiary hospitals appeared to be increasingly directed towards less severe diseases relative to the severe chronic diseases. This finding is consistent with a finding from a prior study, where we observed that while the total number of healthcare contacts in Danish children under five years of age remained stable, the number of contacts with specialist care increased during 1999–2016^19^. In a world with an increasing need for health care (due to demographic changes) and a limited number of healthcare professionals, such findings warrant reflection and underline the need for prioritization.

Several irregularities were observed in the number of contacts over the study period 2000–2018. Many of these developments could be attributed to changes in administration and registration of health services. E.g., the drop in admissions at Rigshospitalet in 2017 is hypothesized to be partially due to a new electronic journaling system implemented during 2016, which shifted the task of entering information into medical records from medical secretaries to physicians, leading to a reduction in hospital activity and most likely also causing some contacts to be missing in the DNPR^31^. Organizational and administrative priorities, like frequent admission of children and adolescents with certain diagnoses such as cancer, are visible and may warrant discussion of prioritization. The Supplementary Information (pages 20–21) contains additional hypotheses regarding the observed changes. The results enable comparisons of 0–17-year-old patients at the four Danish tertiary hospitals. The numbers of contacts were similar at Rigshospitalet, Odense University Hospital, and Aarhus University Hospital, while Aalborg University Hospital had fewer contacts. All hospitals had patients with severe medical diagnoses, such as leukemia and disorders related to prematurity, showing that all four hospitals provided specialized pediatric treatment. However, descriptors of pediatric contacts varied across the four hospitals in terms of the distribution of diagnoses, the percentage of contacts with a severe chronic disease, and the hospitals’ uptake areas. Many diagnoses were ever present, but due to national centralization of certain treatments, some diagnoses were more prevalent at particular hospitals. For instance, all Danish cleft lip and palate patients were referred to Rigshospitalet during the whole follow-up period, and since 2016 treatment of congenital heart disease has also been centralized at Rigshospitalet. In contrast, very few contacts with type 1 diabetes were seen at Rigshospitalet since treatment of diabetes was centralized at another pediatric department in the Capital Region. Both a high percentage of contacts with a severe chronic disease and a high percentage of patients living far away from the hospital indicate a high degree of specialization at Rigshospitalet. However, the latter probably also reflected the Capital Region’s proximity to Region Zealand, which was the only Region without a tertiary hospital. Notably, Eastern Denmark (the Capital Region and Region Zealand) accounted for 44.1% of the person-time spent under 18 years old in Denmark 2000–2018. Aarhus University Hospital had the second highest percentages of contacts with a severe chronic disease and patients from other Regions, followed by Odense University Hospital and Aalborg University Hospital. This ordering of the hospitals regarding severe chronic diseases and residence was the same for in- and outpatients.

Using national register data, it was possible to identify all contacts with tertiary hospitals in Denmark from 2000 to 2018 for individuals 0–17 years old. The rich level of details recorded in the Danish registers enabled a thorough description of the contacts and patients, including information on diagnoses and residence. Still, the data has limitations, including seemingly missing visit dates and the sensitivity to administrative change. Some outpatient records did not have visit dates registered. For these records the start date was counted as the sole visit date. Most records lasted one day, but many lasted at least 31 days (see the Supplementary Information page 5). For records lasting over a month, this assumption is dubious, and it thus seems likely that some visit dates are missing. A large proportion of contacts, in particular outpatient visits, had a main diagnosis of medical observation with no secondary diagnoses to elaborate. This is a weakness of the data source. Though it may be indicative of a high number of contacts not related to any specific conditions requiring treatment, it may also indicate insufficient coding practices. Expanding the study period beyond 2018 requires the integration of two DNPR versions, as a new version was introduced in 2019. The new DNPR does not contain direct information regarding patient-type, leaving it to the researcher to define types of contacts. Suggestions and guidelines for this classification have been proposed^32,33^. This challenge is underlined by the observed effects of administrative changes during 2000–2018 found in the present study.

The results of the study pertain to healthcare activities from the *hospitals’ point-of-view*. While this is appropriate for ascertaining activity at tertiary hospitals, it does not extend to the individual patient-level. Exploring tertiary hospital outcomes and mortality for individuals in a cohort design would provide additional insight.

To understand the impact of organizational differences in tertiary pediatric healthcare and facilitate improvement, it is a prerequisite to understand the current healthcare organization in detail. The present study provided a richly detailed description of tertiary pediatric contacts in Denmark over a 19-year period. Disparities in the severity of pediatric contacts were observed across the four tertiary hospitals even though demographics and healthcare utilization in the five Danish Regions were previously found to be comparable^34^. This knowledge is crucial when comparing pediatric contacts and outcomes at different tertiary hospitals in Denmark. For instance, a naïve comparison of mortality would likely favor hospitals treating less severe conditions. Also, identifying proper control groups to gauge the effect of organizational interventions at a tertiary pediatric hospital is not trivial. As many countries’ populations grow more elderly, the resulting rise in need for health services along with the limited number of healthcare providers is a global challenge^35^. Good prioritization and evaluation of innovative organizational interventions demand the organizational insight provided by the present study.

### Supplementary Information

Supplementary Information.

## Data Availability

The data that support the findings of this study are available from Statistics Denmark, but restrictions apply to the availability of these data, which were used under license for the current study, and so are not publicly available. For help accessing data with permission of Statistics Denmark, contact the corresponding author.
