# Supplementary material for: Pediatric health service utilization at tertiary hospitals in Denmark 2000–2018
Source: Sci Rep. 2024 Jun 6;14:12999. doi: 10.1038/s41598-024-63853-8 (PMC11156864; doi:10.1038/s41598-024-63853-8)
Supplement: Supplementary file 1 — Supplementary Information. [file 41598_2024_63853_MOESM1_ESM.pdf]

Supplementary Information

Pediatric health service utilization at tertiary hospitals in Denmark 2000-2018

Pi Vejsig Madsen, Andreas Jensen, Frank Eriksson, and Lone Graff Stensballe

Table of Contents

Supplementary Methods: Literature Search 2
Supplementary Methods: Migration Dates 3
Supplementary Methods: Hospital and Department Codes 3
Supplementary Methods: Admissions and Outpatient Visits 5
Supplementary Methods: Categorization of Birth and Psychiatric Diagnoses 6
Supplementary Methods: Definition of Severe Chronic Disease 7
Supplementary Note: Background Population 8
Supplementary Note: Unique Patients Excluded 10
Supplementary Note: Acute Outpatient Visits 10
Supplementary Note: Admissions with Only Birth Diagnoses 13
Supplementary Note: Contacts with Public Somatic Hospitals 14
Supplementary Discussion: Changes Over Time and Administrative Changes 20
Supplementary Tables – Contacts at Secondary Hospitals 22
Supplementary Figures and Tables – Inpatients at Tertiary Hospitals 23
Supplementary Figures and Tables – Outpatients at Tertiary Hospitals 39
References 52

Supplementary Methods: Literature Search

The initial literature search aimed to find literature with numeric descriptors of tertiary pediatric healthcare organization at a national level with an emphasis on European countries.

The starting point was a search on PubMed (with language restricted to English and Danish) for the phrases:

"organization tertiary pediatric health service Europe"

"organization tertiary pediatric health service European"

"organization tertiary pediatric healthcare Europe"

All results were screened by title, and seemingly relevant articles were checked for tables and figures presenting quantitative descriptors of pediatric healthcare. Articles regarding pediatric emergency care were not considered. This search helped identify literature describing pediatric healthcare at the national level in Austria,[1] Poland,[2] and Romania.[3] The search was repeated on PubMed with British English spelling ("organisation" and "paediatric"), yielding the same three articles selected above. No additional results were included. Many pertained to exclusively one hospital and were omitted for this reason. The search was also repeated on PubMed without the European part of the three search phrases. This led to a paper regarding nationwide pediatric healthcare in China, which included several hospital statistics.[4]

Snowballing from the three papers on Austria, Poland, and Romania, it was discovered that they were part of two series initiated by the European Pediatric Association-Union of National European Pediatric Societies and Associations, which aimed to describe strengths and weaknesses of the organization of pediatric healthcare in European countries, along with highlighting diversity between countries.[5,6] All articles in the two special issues were screened for statistics regarding organization of pediatric health services. Articles with only numbers regarding demographics, child mortality, vaccination rates, or general healthcare not specific to pediatrics were not included. In addition, attention was restricted to countries within the European Economic Area. Denmark was included due to the focus of the present study. Articles regarding Cyprus,[7] the Czech Republic,[8] Denmark,[9] Hungary,[10] Ireland,[11] Italy,[12] Slovenia,[13] Switzerland,[14] and eight small European countries (Andorra, Cyprus, Iceland, Luxembourg, Malta, Monaco, Montenegro, and San Marino)[15] were selected.

Since the subject matter overlaps management as well as medicine, additional searches were done on interdisciplinary databases. A search on Scopus for *"organization tertiary pediatric health service Europe"* yielded many hits. To vary the screened literature, results were sorted in reverse chronological order (the search was done on October 12, 2023). Most, but not all results, were screened by title. Promising titles were screened for relevant statistics. This search gave more results regarding *tertiary* hospitals, but many were excluded due to focus on emergency units or lack of numeric data. A recent paper comparing neonatal care for preterm births between regions in the five Nordic countries was found.[16]

Finally, Web of Science was searched using the phrases:

"organization tertiary pediatric health service Europe"

"organization tertiary pediatric health service European"

"organization tertiary pediatric healthcare Europe"

"organization tertiary pediatric health service"

This gave a manageable amount of hits, so all results were screened by title. In this manner (using the fourth search string) an article comparing health services for preterm births in 11 high income countries (Australia, Canada, Finland, Israel, Italy, Japan, New Zealand, Spain, Sweden, Switzerland, and the United Kingdom)

was found.[17] Other results were screened and found to contain numeric descriptors but excluded for focusing exclusively one single units or hospitals.

Supplementary Methods: Migration Dates

Repeated migration codes in the Danish migration register, such as two immigrations without an intermediate emigration, were dropped such that only the first date was kept.

Supplementary Methods: Hospital and Department Codes

Hospitals and hospital departments were identified using the hospital-code section of the Danish Medical Classification System, referred to as SHAK (Danish: Sygehus-afdelingsklassifikation).[18]

Supplementary Table S1 shows the hospital id-codes used to define the four tertiary hospitals. Note that Aarhus University Hospital was a composite of three id-codes: the current one along with the codes of two predecessors. Supplementary Table S2 defines pediatric departments at the four tertiary hospitals during 2000-2018.

Supplementary Table S1. Tertiary hospitals (2000-2018)

Hospital name	Included under the current name	Danish hospital ID (SHAK)
Rigshospitalet	Rigshospitalet	1301
Odense University Hospital	Odense University Hospital	4202
Aarhus University Hospital	Aarhus University Hospital	6620
Skejby University Hospital	Aarhus University Hospital	7026
Aarhus Hospital	Aarhus University Hospital	7003
Aalborg University Hospital (previously Aalborg Hospital)	Aalborg University Hospital	8001

Supplementary Table S2. Pediatric departments (2000-2018) at the four tertiary hospitals

Hospital	Department name	Danish hospital department ID (SHAK)
Rigshospitalet	Department of Pediatrics and Adolescent Medicine	130132
Rigshospitalet	Department of Neonatology	130147
Odense University Hospital	Department of Pediatrics and Adolescent Medicine	420225
Aarhus University Hospital	Department of Pediatrics and Adolescent Medicine	662024
Aarhus University Hospital (then, Skejby Hospital)	Department of Pediatrics	702624
Aalborg University Hospital	Department of Pediatrics and Adolescent Medicine	800125

Contacts with public somatic hospitals that are not tertiary (referred to as secondary hospitals) were also considered. Many hospitals changed names or were closed during the study period 2000-2018. The following SHAK codes were used to define secondary hospitals, note that many of them are no longer in use. All Danish names used during 2000-2018 listed in reverse chronological order. Secondary hospital SHAK codes:

- 1309: Bispebjerg og Frederiksberg Hospitaler. Bispebjerg Hospital.
- 1330: Amager og Hvidovre Hospital. Hvidovre Hospital.
- 1351: Amager Hospital.
- 1401: Frederiksberg Hospital.
- 1501: Gentofte Hospital. Kbh. Amts Sygehus i Gentofte.
- 1502: Glostrup Hospital. Kbh. Amts Sygehus i Glostrup.
- 1507: Steno Diabetes Center.
- 1516: Herlev og Gentofte Hospital. Herlev Hospital. Kbh. Amts Sygehus i Herlev.
- 2000: Hospitalerne i Nordsjælland. Frederiksborg Amts Sundhedsvæsen.
- 2501: Roskilde Sygehus. Roskilde Amts Sygehus, Roskilde. Amtssygehuset i Roskilde.
- 2502: Køge Sygehus. Roskilde Amts Sygehus, Køge. Amtssygehuset i Køge.
- 3000: Sygehus Vestsjælland.
- 3001: Holbæk Sygehus. Centralsygehuset i Holbæk.
- 3002: Slagelse Sygehus. Centralsygehuset i Slagelse.

- 3003: Kalundborg Sygehus.
- 3004: Ringsted Sygehus.
- 3500: Storstrømmens Sygehus.
- 3501: Centralsygehuset i Næstved.
- 3502: Centralsygehuset i Nykøbing F.
- 3503: Amtssygehuset i Fakse.
- 3505: Amtssygehuset i Nakskov.
- 3506: Amtssygehuset i Stege.
- 3800: Region Sjællands Sygehusvæsen.
- 4001: Bornholms Hospital. Bornholms Centralsygehus.
- 4212: OUH Svendborg Sygehus. Sygehus Fyn.
- 5000: Sygehus Sønderjylland.
- 5001: Sygehus Sønderjylland, Sønderborg. Sønderborg Sygehus.
- 5002: Sygehus Sønderjylland, Haderslev. Haderslev Sygehus.
- 5003: Sygehus Sønderjylland, Tønder. Tønder Sygehus.
- 5004: Sygehus Sønderjylland, Aabenraa. Aabenraa Sygehus.
- 5501: Esbjerg Sygehus Grindsted Sygehus. Sydvestjysk Sygehus. Esbjerg Centralsygehus.
- 5502: Ribe Sygehus.
- 5503: Varde Sygehus.
- 5504: Grindsted Sygehus.
- 5505: Brørup Sygehus.
- 6002: Brædstrup Sygehus.
- 6003: Fredericia Sygehus.
- 6004: Give Sygehus.
- 6006: Regionshospitalet Horsens. Regionshospitalet Horsens og Brædstrup. Regionshospitalet Horsens, Brædstrup og Odder. Horsens Sygehus.
- 6007: Kolding Sygehus. Fredericia og Kolding Sygehuse.
- 6008: Vejle Sygehus.
- 6013: Friklinikken Region Syddanmark (Grindsted). Friklinikken Region Syddanmark (Give). De Vestdanske Friklinikker, Give.
- 6014: De Vestdanske Friklinikker, Brædstrup. Friklinikken i Brædstrup.
- 6501: Regionshospitalet Holstebro. Holstebro Sygehus. Holstebro Centralsygehus.
- 6502: Regionshospitalet Herning. Herning Sygehus. Herning Centralsygehus.
- 6503: Regionshospitalet Tarm. Tarm Sygehus.
- 6504: Regionshospitalet Ringkøbing. Ringkøbing Sygehus.
- 6505: Regionshospitalet Lemvig. Lemvig Sygehus.
- 6506: Hvidbjerg Amtssygehus.
- 6630: Hospitalsenhed Midt.
- 6650: Hospitalsenheden Vest.
- 7002: Regionshospitalet Silkeborg. Silkeborg Centralsygehus.
- 7004: Århus Amtssygehus.
- 7005: Regionshospitalet Randers. Randers Centralsygehus.
- 7007: Marselisborg Hospital.
- 7008: Odder Sygehus. Odder Centralsygehus.
- 7009: Regionshospitalet Grenaa. Grenaa Sygehus. Grenaa Centralsygehus.
- 7010: Regionshospitalet Hammel Neurocenter. Hammel Neurocenter.
- 7013: Samsø Sundheds- og Akuthus. Samsø Syge- og sundhedshus.
- 7601: Regionshospitalet Viborg, Skive. Regionshospitalet Viborg, Skive og Kjellerup. Sygehus Viborg. Viborg-Kjellerup Sygehus.
- 7602: Skive Sygehus.
- 7603: Aalborg Universitetshospital, Thisted. Regionshospital Nordjylland Thy. Sygehus Thy - Mors. Sygehus NORD, Nykøbing-Thisted. Nykøbing/Thisted Sygehus.
- 8003: Regionshospital Nordjylland Vendsyssel. Sygehus Vendsyssel. Hjørring-Brønderslev Sygehus.
- 8004: Hobro-Terndrup Sygehus.
- 8005: Sygehus Himmerland. Farsø Sygehus.
- 8007: Dronninglund Sygehus.
- 8008: Frederikshavn-Skagen Sygehus.
- 8009: Brovst Sygehus.
- 8050: Anæstesisektor Nordjylland.

Supplementary Methods: Admissions and Outpatient Visits

Patients were categorized as inpatients or elective outpatients using the patient-type and urgency variables in the Danish National Patient Registry (DNPR). Inpatients were defined as records coded “full-time” or “hospitalized”. Outpatients were defined as records coded “part-time” or “ambulatory”. Acute outpatients were identified as outpatients marked as urgent or records with patient-type “emergency room”.

Admissions were consolidated by setting the second admission date to the previous discharge date for overlapping records. If necessary, the discharge date was also carried forward such that admission always preceded discharge. Adjacent inpatient records, i.e., a record starting the same day the previous ended, were consolidated into one joint admission.

The included outpatient visit dates were based on the 2000-2018-versions of DNPR, supplemented with visit dates between 2000-2018 found in the 2019-DNPR-table for outpatient records which were ongoing as of December 31, 2018. This is expected to include all visit dates registered in DNPR, since a change to the register’s format was implemented during 2019, necessitating the closure of all active records during 2019. Outpatient visits that satisfied the inclusion and exclusion criteria, but did not have visit dates, were included in the study with the start of the record acting as the sole visit date. Of the 1,901,149 selected outpatient records, 146,126 (7.7%) did not have visit dates available. Of these outpatient records without visit dates, 53.1% lasted one day, while 40.9% lasted at least seven days, and 31.9% lasted at least 31 days. The median length was 1 day (interquartile range: 1-85 days).

We chose to focus on admissions and outpatient visits primarily due to their interpretability. In - and outpatient records were considered in earlier phases of the study. In the present final form, adjacent inpatient records were consolidated into admissions with 0.89 admissions per inpatient record. Outpatient records were found to be more sensitive towards administrative changes, in the sense that many anomalies seen in the selected contacts were exaggerated when considering records (compare Supplementary Figure S1 to Figure 4).

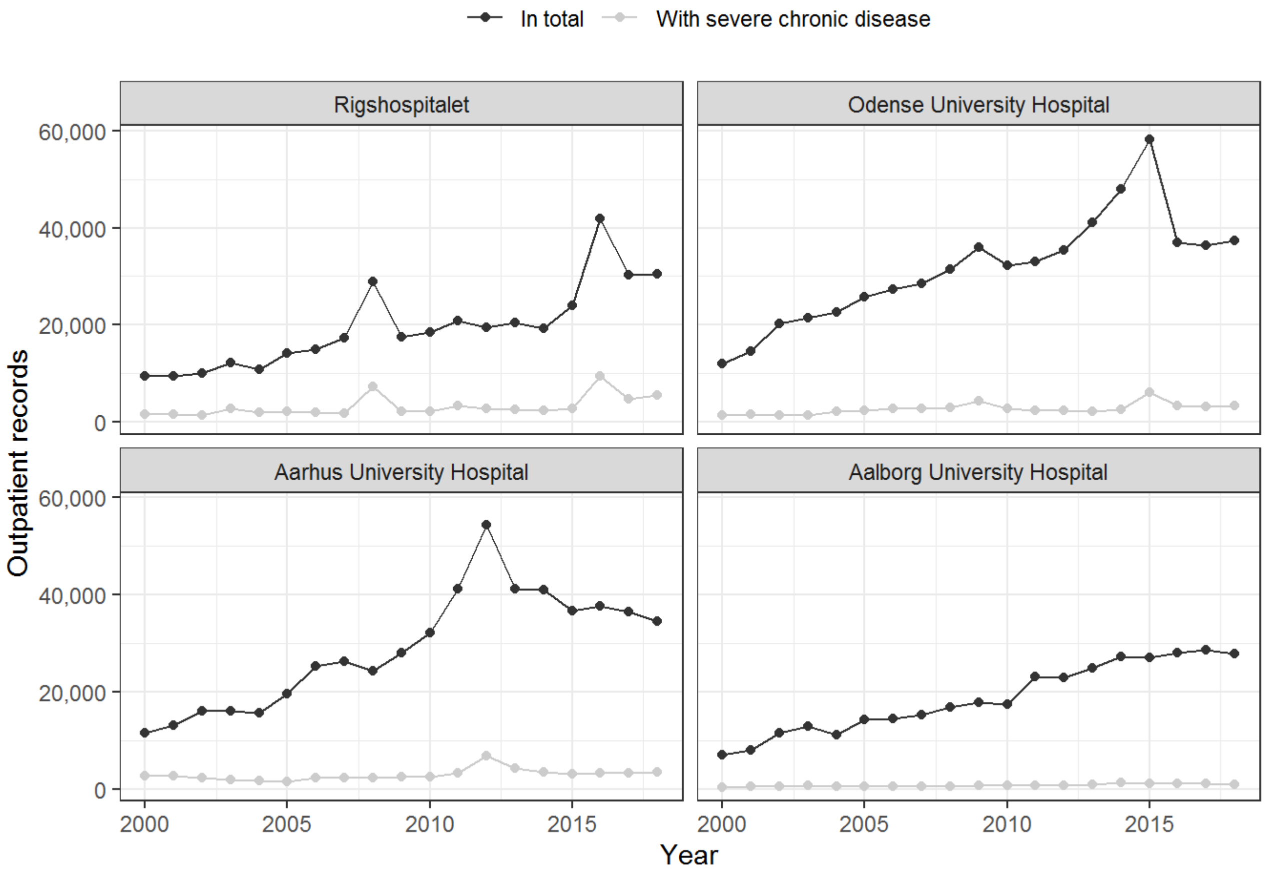

Supplementary Figure S1. Outpatient records starting 2000-2018, applying the same inclusion/exclusion-criteria as for visits

Supplementary Methods: Categorization of Birth and Psychiatric Diagnoses

Birth diagnoses were defined as diagnoses starting with DZ38, DZ308, DU, DV, or DO in the Danish Medical Classification System, which is an augmentation of the ICD-10 system adding the prefix ‘D’ to the ICD-10 code. Psychiatric diagnoses were defined as any diagnosis in ICD-10-chapter V.

Supplementary Methods: Definition of Severe Chronic Disease

Supplementary Table S3. Diagnoses (ICD-10 codes) used to define severe chronic disease

**ICD10-codes**	**Explanation**
C00.0-C99.1	Malignant neoplasms
D61.0; D61.3; D61.8-D61.9	Aplastic anemias
D76.2	Hemophagocytic syndrome, infection-associated
D80.0-D82.9	Immunodeficiencies
E10	Insulin-dependent diabetes mellitus
E25	Adrenogenital disorders
E70.0-73.0	Disorders of amino-acid metabolism
E74.0-E84.9	Disorders of metabolism and cystic fibrosis
G12	Spinal muscular atrophy and related syndromes
G31.0; G31.8–G31.9; G37.0–G37.9	Degenerative and demyelinating diseases of nervous system
G40	Epilepsy
G60	Hereditary and idiopathic neuropathy
G70.2	Congenital and developmental myasthenia
G71.0-G71.3	Mitochondrial myopathy
G73.6	Myopathy in metabolic diseases
G80	Cerebral palsy
G81.1; G82.1; G82.4	Spastic conditions
G91	Hydrocephalus
G94.1	Hydrocephalus in neoplastic disease
I12	Hypertensive renal disease without renal failure
I27.0-I27.9	Pulmonary heart disease
I30.0-I52.8	Other forms of heart disease
J44.8	Other specified chronic obstructive pulmonary disease
J84	Other interstitial pulmonary diseases
K21	Gastro-esophageal reflux disease
K50.0–K51.9	Crohn disease [regional enteritis] and ulcerative colitis
K70.0-K77.8	Diseases of liver
K90	Intestinal malabsorption
M30.0-M35.9	Systemic involvement of connective tissue
N03-N05	Nephritic syndrome
N07	Hereditary nephropathy, not elsewhere classified
N13	Obstructive and reflux uropathy
N18.0-N19.9; N25.0-N27.9	Chronic kidney disease
P27	Chronic respiratory disease originating in the perinatal period
P57.0-P57.9	Kernicterus
P91.0-P91.2	Disturbances of cerebral status of newborn
P94.1-P94.9	Disorders of muscle tone of newborn
Q01-Q07	Congenital malformations of the nervous system
Q20.0-Q26.9	Congenital malformations of the circulatory system
Q30.0-Q32.4	Congenital malformations of nose, larynx, trachea, and bronchus
Q33.0-Q33.9	Congenital malformations of lung
Q34.0-Q37.9	Other congenital malformations of respiratory system, cleft lip, and cleft palate
Q39.0-Q45.3	Congenital malformations of upper alimentary tract, intestine, and pancreas
Q60.0-Q64.9	Congenital malformations of the urinary system
Q79.0	Congenital diaphragmatic hernia
Q79.2-Q79.3	Exomphalos and gastroschisis
Q86.0	Fetal alcohol syndrome (dysmorphic)
Q87	Other specified congenital malformation syndromes affecting multiple systems
Q90.0-Q99.9	Chromosomal abnormalities, not elsewhere classified

Supplementary Note: Background Population

To provide context for the number of contacts observed at hospitals in Denmark, we consider the population of children and adolescents living in Denmark. The background population was defined as individuals 0-17 years old living in Denmark during 2000-2018. In total, 2,496,001 unique individuals were part of the background population. They spent a combined 22,620,953 person-years in Denmark while under 18 years old.

The number of unique individuals in the background population each year remained relatively stable throughout the study period, as seen in Supplementary Figure S2. The number of children and adolescents living in Denmark increased year by year from 2000 to 2008, at which point it started to decrease. The figure also shows that the absolute number of unique individuals per year who had an included tertiary hospital contact increased during the study period. The corresponding number are found in Supplementary Table S4. While the number of individuals in the population of 0-17-year-olds in Denmark topped in 2008, the number of individuals with an included tertiary hospital contact continued to rise until 2016.

The amount of person-time spent in Denmark while aged 0-17 years old is presented in Supplementary Figure S3 and Supplementary Table S5. It exhibits a pattern similar to the absolute number of individuals at risk per year.

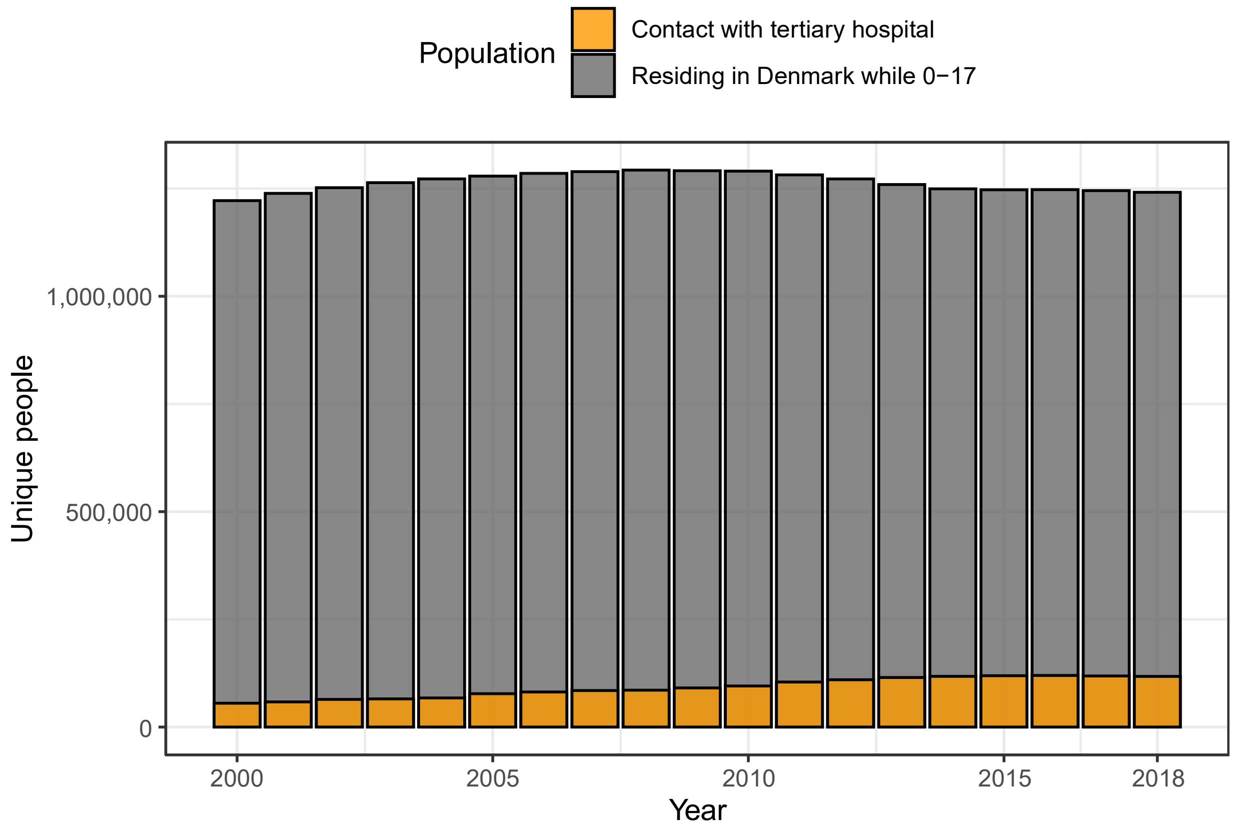

Supplementary Figure S2. Unique individuals per year who at some point during said year lived in Denmark while under 18 years old, and the subgroup of this population who had at least one included contact with a tertiary hospital during the year

Supplementary Table S4. Unique individuals per year who at some point during said year lived in Denmark while under 18 years old, and the subgroup of this population who had at least one included contact with a tertiary hospital during the year

Year	Individuals in background population	Individuals with an included tertiary contact
2000	1,221,989	55,356
2001	1,238,858	58,392
2002	1,252,045	64,215
2003	1,263,700	65,373
2004	1,272,441	67,573
2005	1,278,904	77,414
2006	1,285,272	81,357
2007	1,289,278	84,725
2008	1,292,879	85,594
2009	1,291,407	90,867
2010	1,290,436	95,288
2011	1,281,721	104,472
2012	1,272,339	109,843
2013	1,259,317	115,026
2014	1,249,280	117,752
2015	1,247,207	119,170
2016	1,247,486	119,779
2017	1,245,231	118,514
2018	1,241,316	117,615

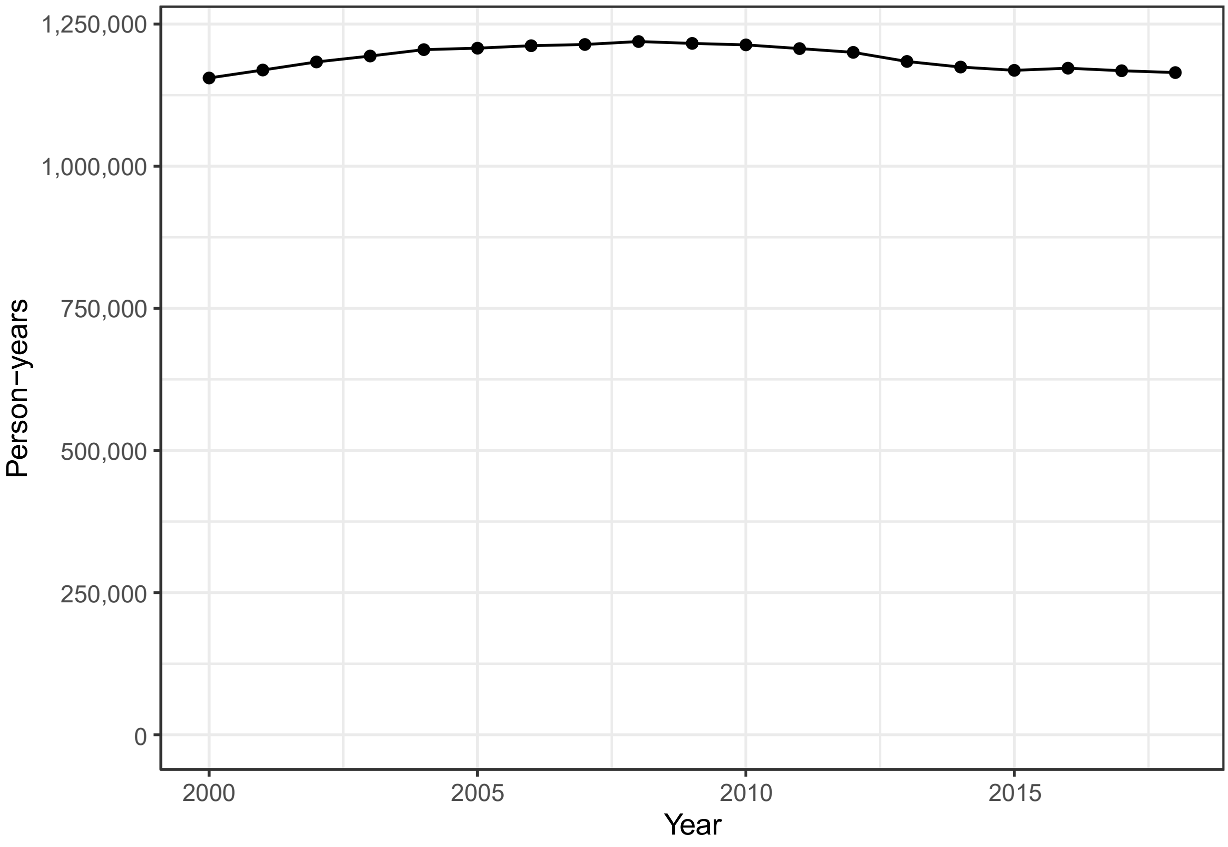

Supplementary Figure S3. Person-time (in years) per calendar year spent in Denmark while under 18 years old

Supplementary Table S5. Person-time (in years) per calendar year spent in Denmark while under 18 years old

Year	Person-years
2000	1,155,168
2001	1,169,159
2002	1,183,367
2003	1,193,787
2004	1,205,010
2005	1,207,561
2006	1,211,931
2007	1,214,085
2008	1,219,266
2009	1,215,978
2010	1,213,384
2011	1,206,858
2012	1,200,222
2013	1,184,216
2014	1,174,383
2015	1,168,598
2016	1,172,374
2017	1,167,900
2018	1,164,682

Supplementary Note: Unique Patients Excluded

The selected contacts with the four tertiary hospitals were from 740,263 unique individuals. By omitting acute outpatients, 78,618 individuals were excluded. By omitting admissions with only birth diagnoses, 25,239 individuals (of which 5,630 individuals were also part of the group excluded as acute outpatients) were excluded. Thus, 98,227 individuals were excluded due to these exclusion criteria.

Supplementary Note: Acute Outpatient Visits

A total of 733,071 acute outpatient visits for 353,236 unique 0-17-year-old patients occurred at the four tertiary hospitals during 2000-2018. A low percentage, only 1.1%, of these visits included a diagnosis of a severe chronic disease. Most visits, 72.5%, had an external injury (ICD-10-chapter XIX) as the main diagnosis. The percentage varied between hospitals: 48.8% at Rigshospitalet, 78.0% at Odense University Hospital, 77.1% at Aarhus University Hospital, and 64.0% at Aalborg University Hospital.

Compared to the inpatients and elective outpatients, the acute outpatients were older. The median age at time of visit was 9.72 years (interquartile range: 3.87-14.0). The most common age at time of visit was one year (56,417 visits). Supplementary Figure S4 contains the details regarding the overall age of the patients. Patients at Rigshospitalet were generally younger (most common age zero years) compared to the other three hospitals (Supplementary Figure S5).

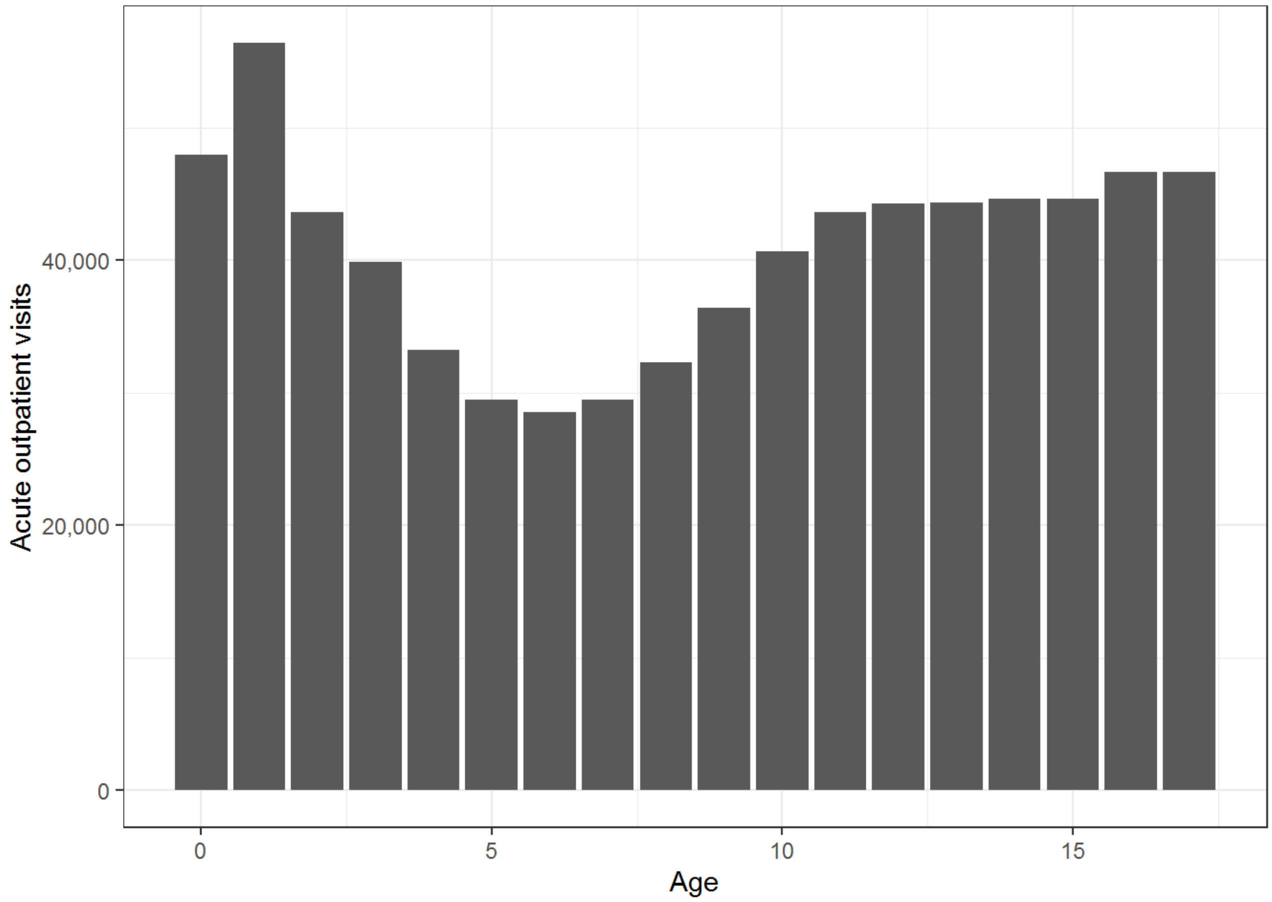

Supplementary Figure S4. Age of patients at time of acute outpatient visits

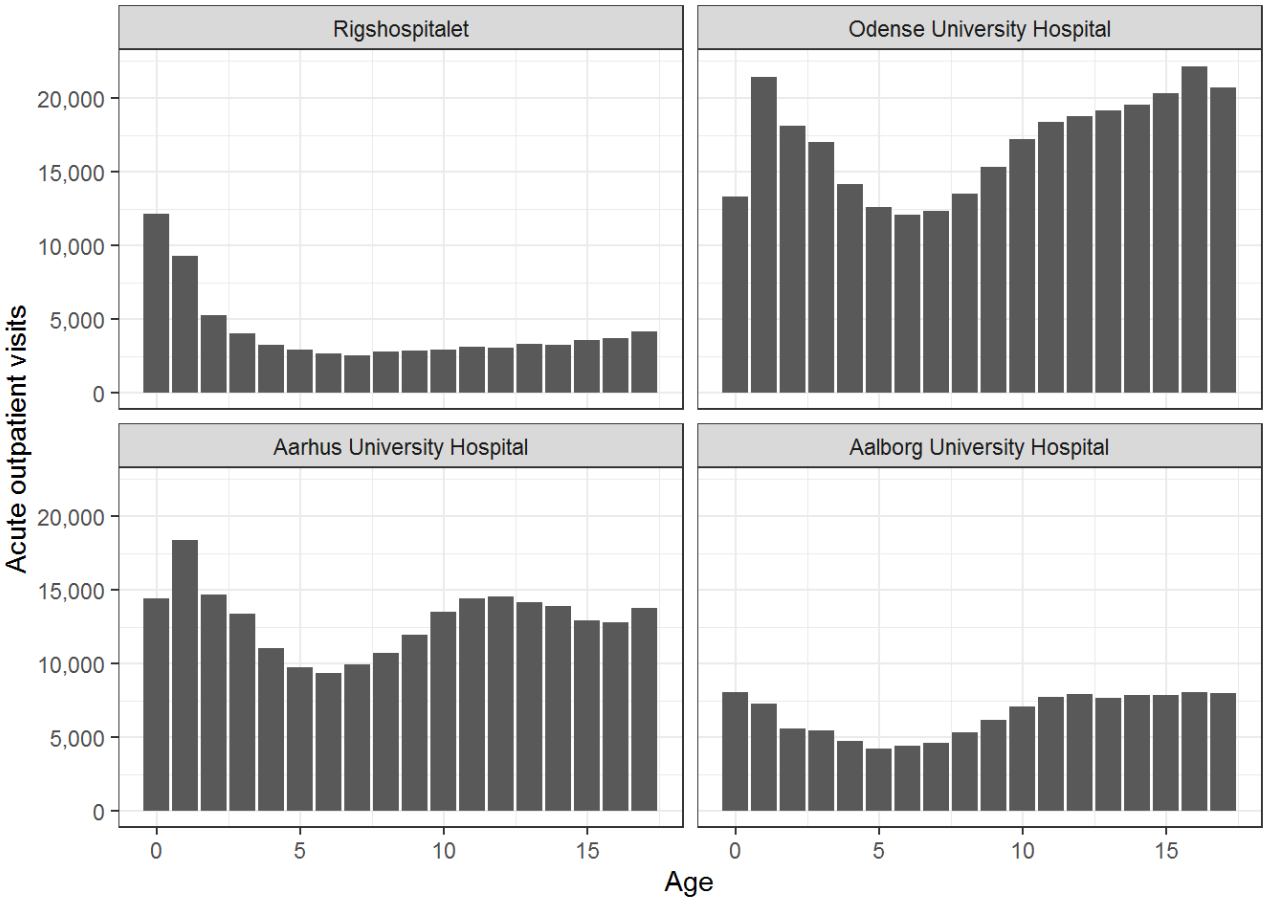

Supplementary Figure S5. Age of patients at time of acute outpatient visits by hospital

Supplementary Figure S6 shows the number of selected acute outpatient visits from 2000 to 2018. The number of visits increased dramatically within all four hospitals starting in the 2010's. Part of this might be explained by a change in the administration of out-of-hours contacts, mandating that patients were referred to emergency rooms. This became a nationwide principal in 2014. On January 1, 2014, the emergency room patient-type in the Danish National Patient Registry was phased out and replaced by coding them as ambulatory patients with an acute referral code. After 2013, an increase in the number of acute outpatient visits in Denmark has also been found among adults (aged 18 and above).[19]

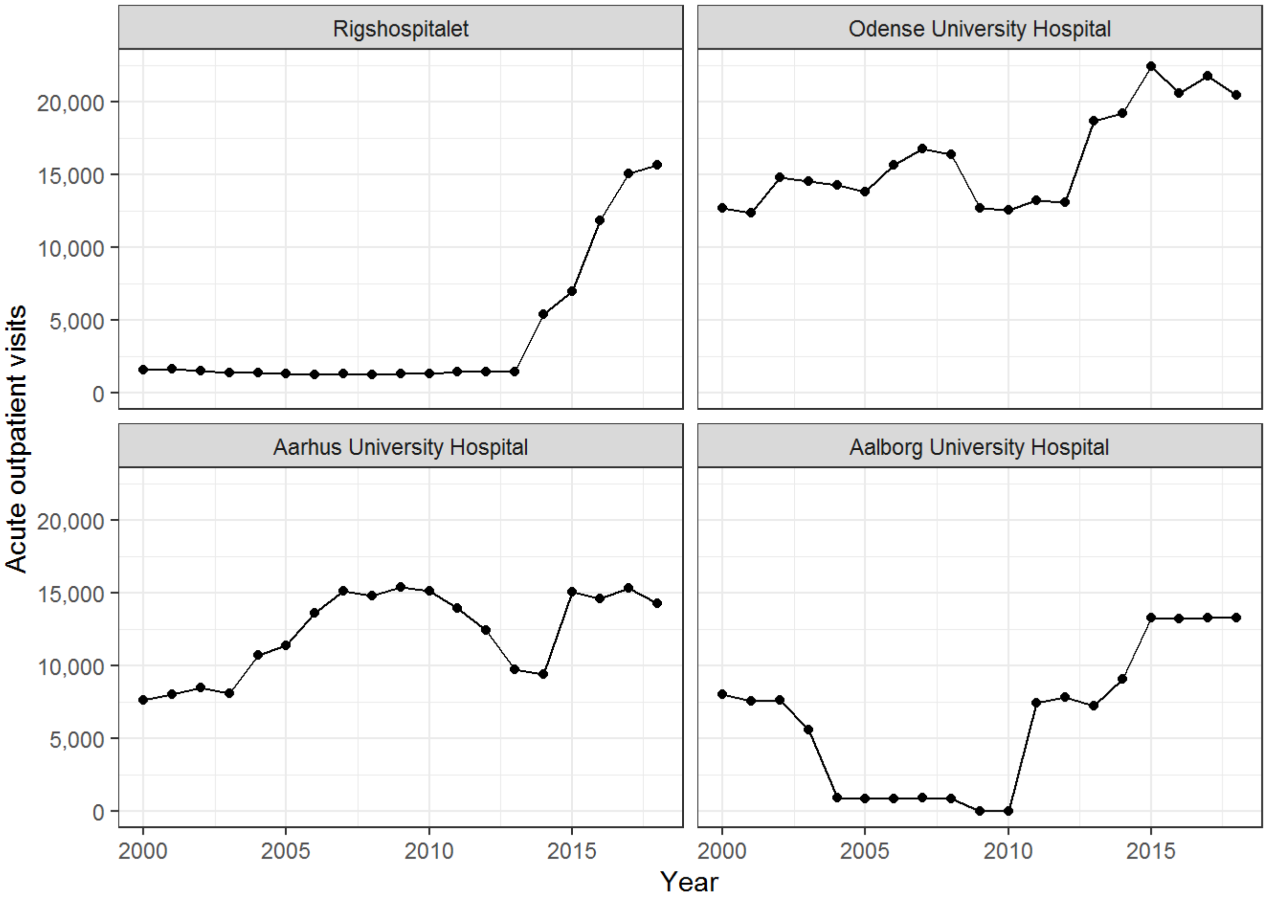

Supplementary Figure S6. Acute outpatient visits from 2000-2018 at the four tertiary hospitals

Supplementary Note: Admissions with Only Birth Diagnoses

During the study period, there were 189,499 admissions at the four tertiary hospitals with only birth diagnoses. The admissions corresponded to 189,436 unique patients. The sex of the admitted patients was approximately equal parts male and female (Supplementary Table S6). Details regarding the development over the period 2000-2018 are found in Supplementary Figure S7.

Supplementary Table S6. Sex of patients admitted with exclusively birth diagnoses. Percentages relative to the hospital

Hospital	Male	Female
Rigshospitalet	29,681 (50.9%)	28,600 (49.1%)
Odense University Hospital	18,056 (49.0%)	18,824 (51.0%)
Aarhus University Hospital	32,380 (50.6%)	31,552 (49.4%)
Aalborg University Hospital	15,129 (49.8%)	15,277 (50.2%)

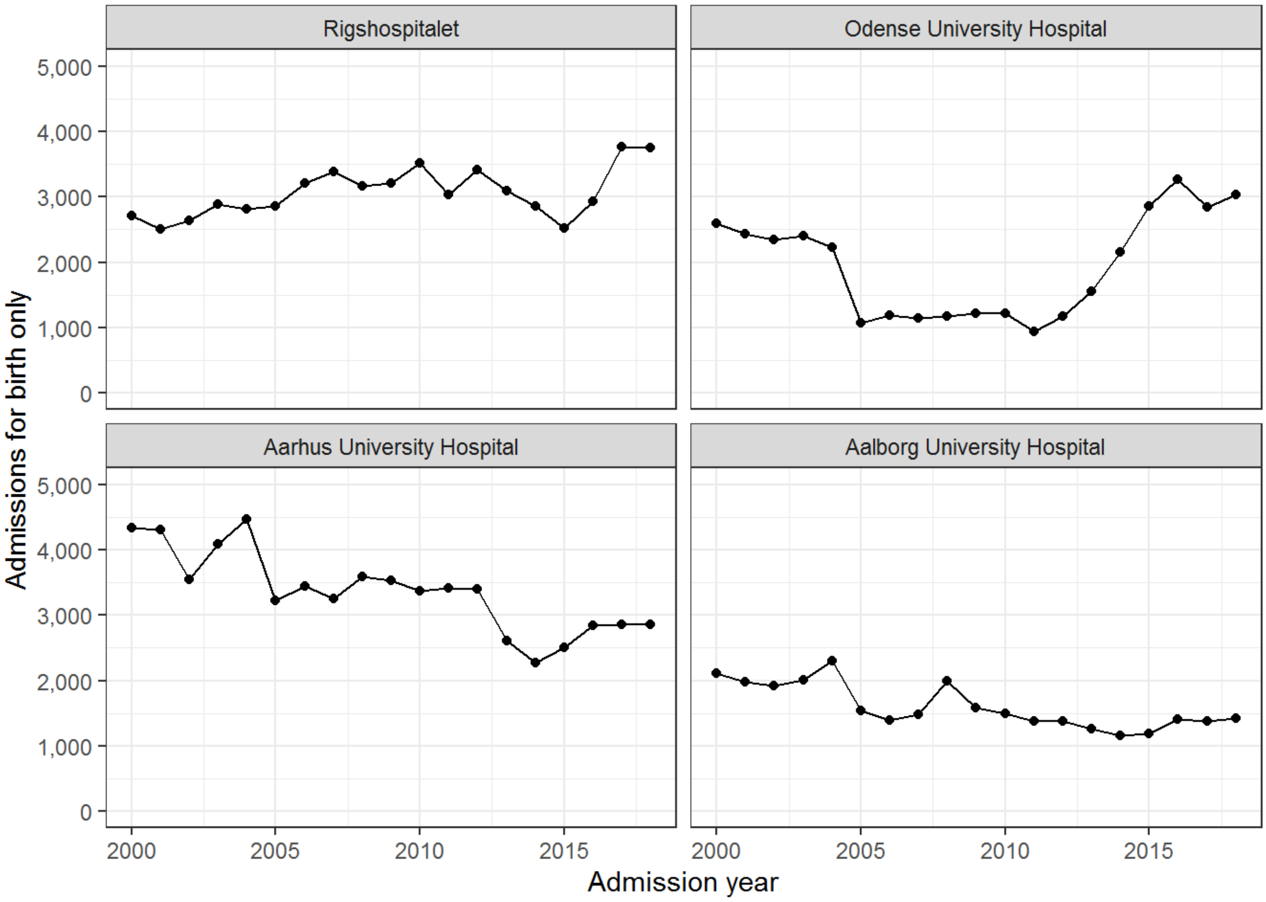

Supplementary Figure S7. Admission for hospitalization with exclusively birth diagnoses between 2000 and 2018 by hospital

Supplementary Note: Contacts with Public Somatic Hospitals

The focal point of the study was tertiary hospitals. A total of 829,562 admissions and 3,932,744 outpatient visits at the tertiary hospital from 740,263 unique patients were included. Employing the same inclusion and exclusion criteria to secondary hospital contacts, a total of 1,971,559 admissions and 7,322,829 outpatient visits were identified. These contacts stemmed from 1,582,945 unique patients, of which 412,320 (55.7% of patients at tertiary hospitals, 26.0% of patients at secondary hospitals) also had a contact with a tertiary hospital. The number of contacts per year for the four tertiary hospitals combined and the secondary hospitals combined are found in Supplementary Table S7 and Supplementary Table S8. These tables correspond in Figure 2. Supplementary Figure S8 shows the incidence rate of contacts at tertiary and secondary hospitals, compare to Figure 2.

When combining all included contacts across tertiary and secondary hospitals, the number of admissions shows a slight initial increase, see Supplementary Figure S9. The number of outpatient visits increased from 2000 to 2012, followed by a slight decrease 2015-2018 (Supplementary Figure S11).

As seen in Supplementary Table S5 and Supplementary Figure S3, the person-time spent living in Denmark while 0-17 years old remained stable throughout 2000-2018. Thus, incidence rate plots of hospital contacts per person-years have a shape exceedingly close to plots of the absolute number of contacts. Supplementary

Figure S10 and Supplementary Figure S12 show respectively the incidence rate for admissions and outpatient visits at public somatic hospitals.

Supplementary Table S7. Admissions at tertiary and secondary hospitals per admission year. Admissions starting prior to 2000 not shown

Year	Tertiary hospitals		Secondary hospitals	
	In total	With severe chronic disease	In total	With severe chronic disease
2000	35,778	10,284	89,899	6,378
2001	38,060	11,135	91,051	6,879
2002	38,765	11,728	89,377	7,192
2003	39,613	11,915	91,176	7,254
2004	40,967	13,395	92,438	7,792
2005	45,132	13,991	100,184	8,227
2006	42,686	12,739	105,081	8,890
2007	42,522	11,707	107,363	9,208
2008	40,787	11,758	104,172	9,079
2009	44,875	12,266	112,122	9,904
2010	49,270	13,835	115,561	10,366
2011	52,360	13,643	111,821	9,778
2012	53,690	14,239	106,492	9,824
2013	54,285	14,251	106,003	10,203
2014	49,193	13,309	107,623	10,370
2015	44,765	12,718	102,163	10,483
2016	43,829	12,316	109,482	9,969
2017	36,932	9,370	114,719	8,237
2018	35,786	9,530	114,208	6,782

Supplementary Table S8. Outpatient visits at tertiary and secondary hospitals per year. The included outpatient records starting prior to 2000 without visit dates are not shown

Year	Tertiary hospitals		Secondary hospitals	
	In total	With severe chronic disease	In total	With severe chronic disease
2000	109,998	29,456	241,283	27,900
2001	118,040	30,930	257,164	29,309
2002	133,562	30,870	319,674	32,062
2003	146,774	32,998	315,585	33,326
2004	155,340	36,689	338,145	37,970
2005	170,773	38,321	380,857	39,519
2006	188,286	42,220	397,558	40,812
2007	192,273	40,623	413,484	42,657
2008	191,287	42,031	404,217	42,279
2009	206,803	44,445	434,230	42,591
2010	216,350	46,648	442,276	42,472
2011	237,892	47,978	443,661	43,424
2012	250,860	49,096	441,677	44,329
2013	262,993	53,992	429,888	43,791
2014	272,928	53,943	431,100	42,851
2015	271,720	51,318	426,235	42,331
2016	265,874	51,877	414,313	39,918
2017	271,203	51,742	400,686	39,750
2018	269,210	51,555	389,914	36,486

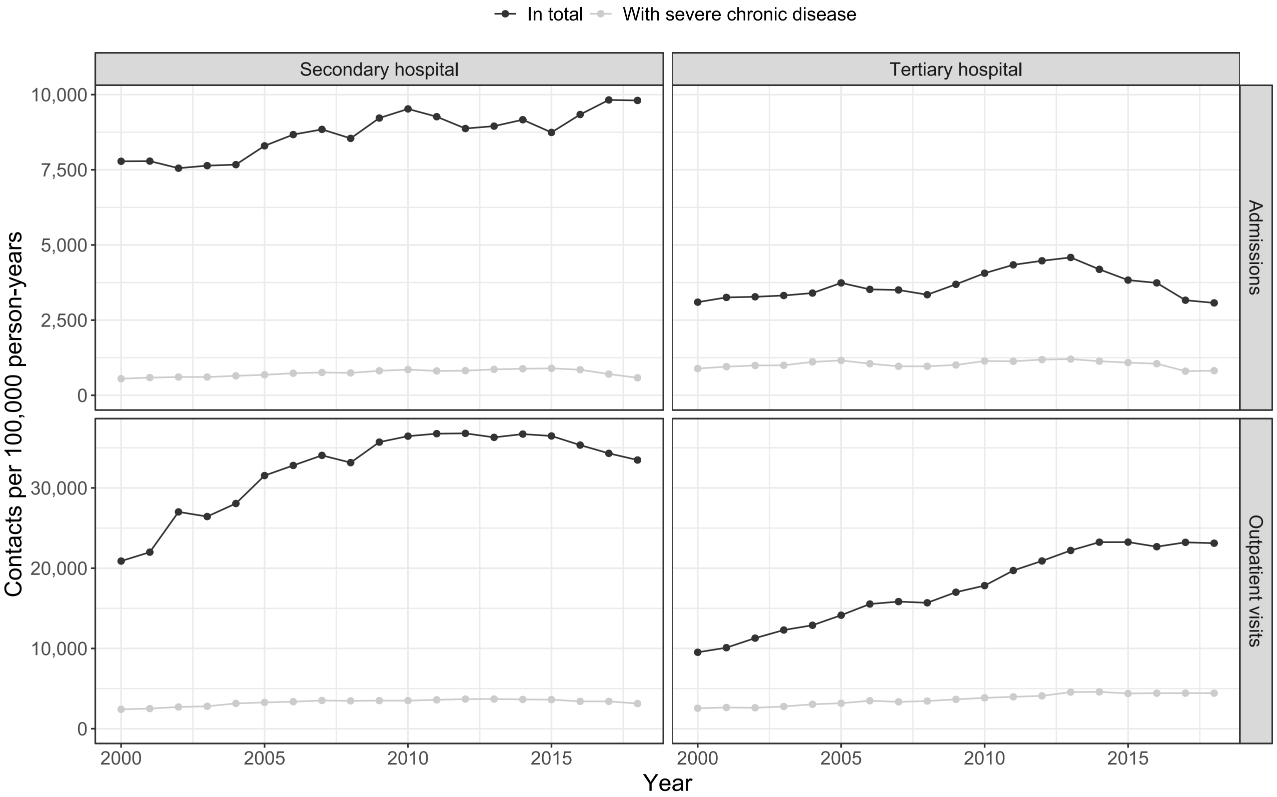

Supplementary Figure S8. Incidence rates of admissions and outpatient visits at secondary and tertiary hospitals. See Supplementary Table S5 for the person-time. Figure 2 shows the corresponding absolute numbers

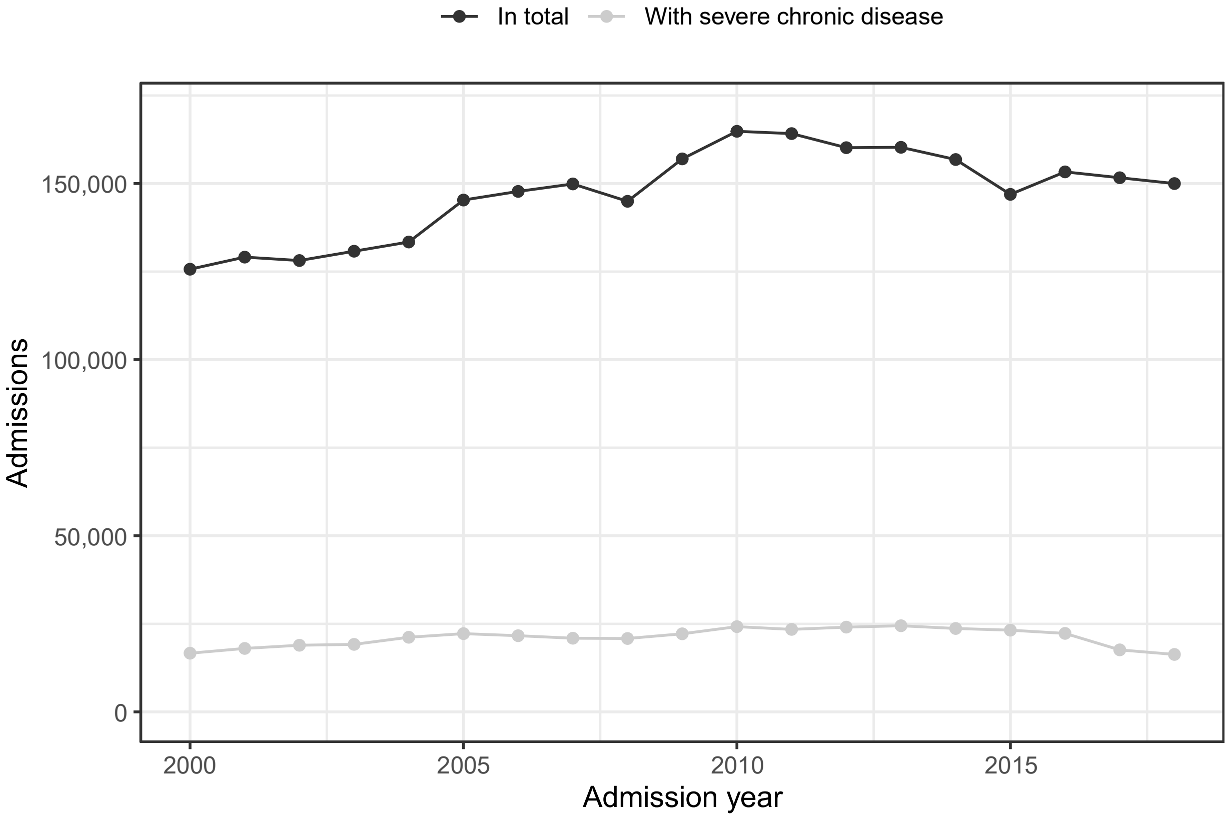

Supplementary Figure S9. Admissions at public somatic hospitals, i.e., tertiary and secondary hospitals combined, by year of admission. Admissions starting prior to 2000 not shown

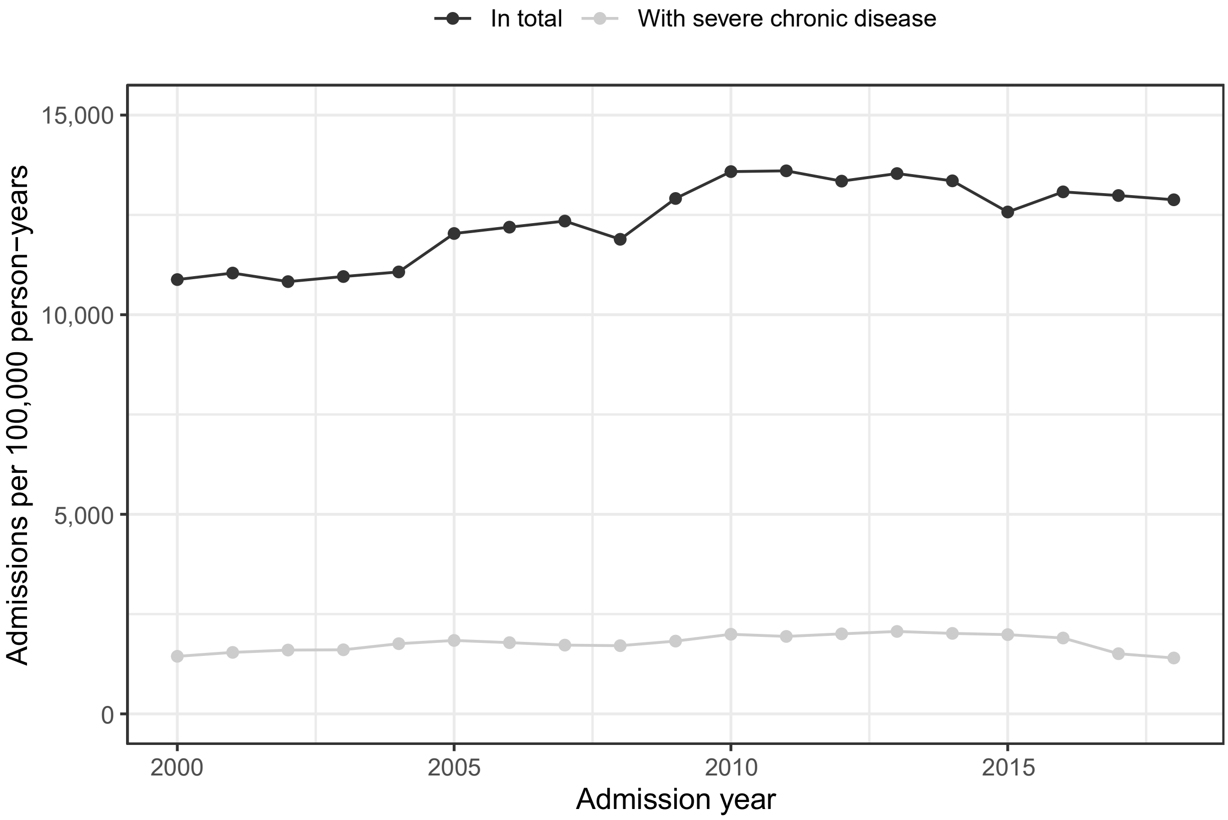

Supplementary Figure S10. Incidence rate of admissions at public somatic hospitals, i.e., tertiary and secondary hospitals combined, by year of admission. Admissions starting prior to 2000 not shown. See Supplementary Table S5 for the person-time

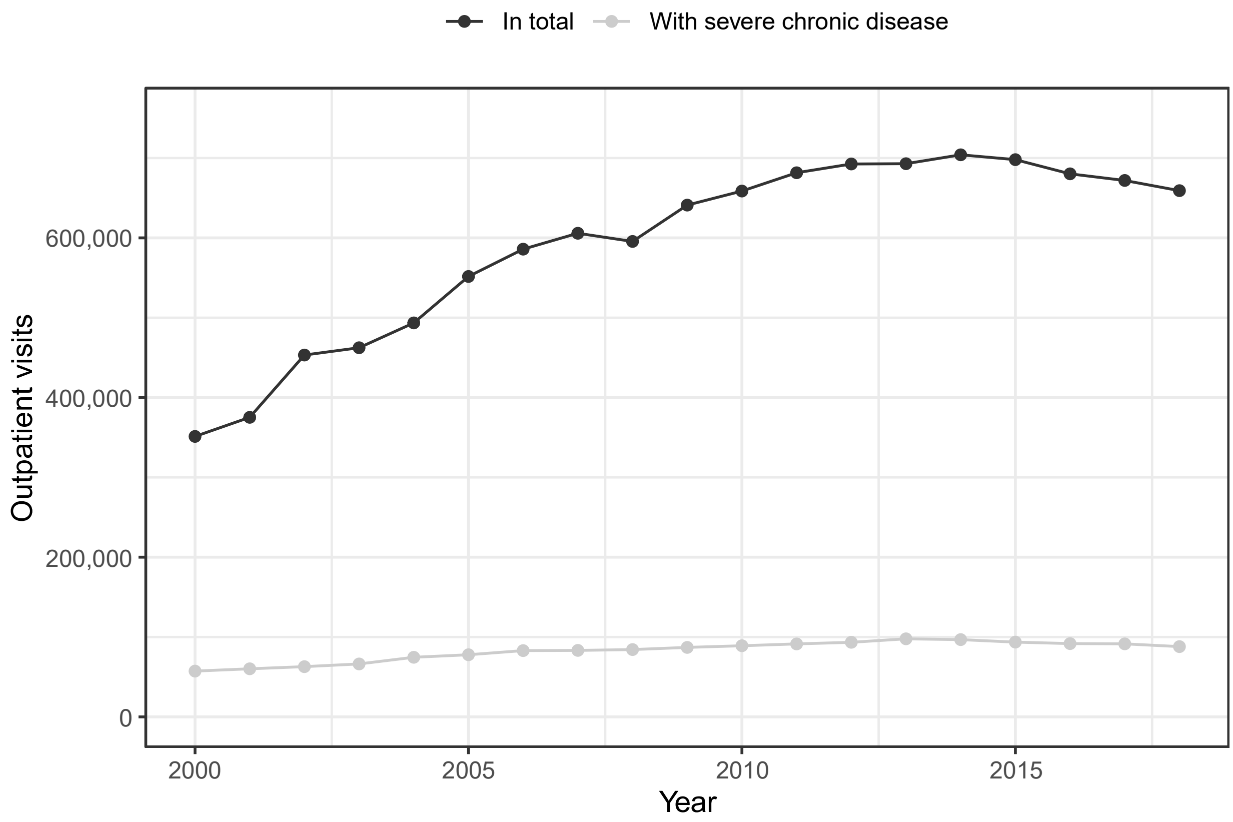

Supplementary Figure S11. Outpatient visits at public somatic hospitals, i.e., tertiary and secondary hospitals combined, by year. The included outpatient records starting prior to 2000 without visit dates are not shown

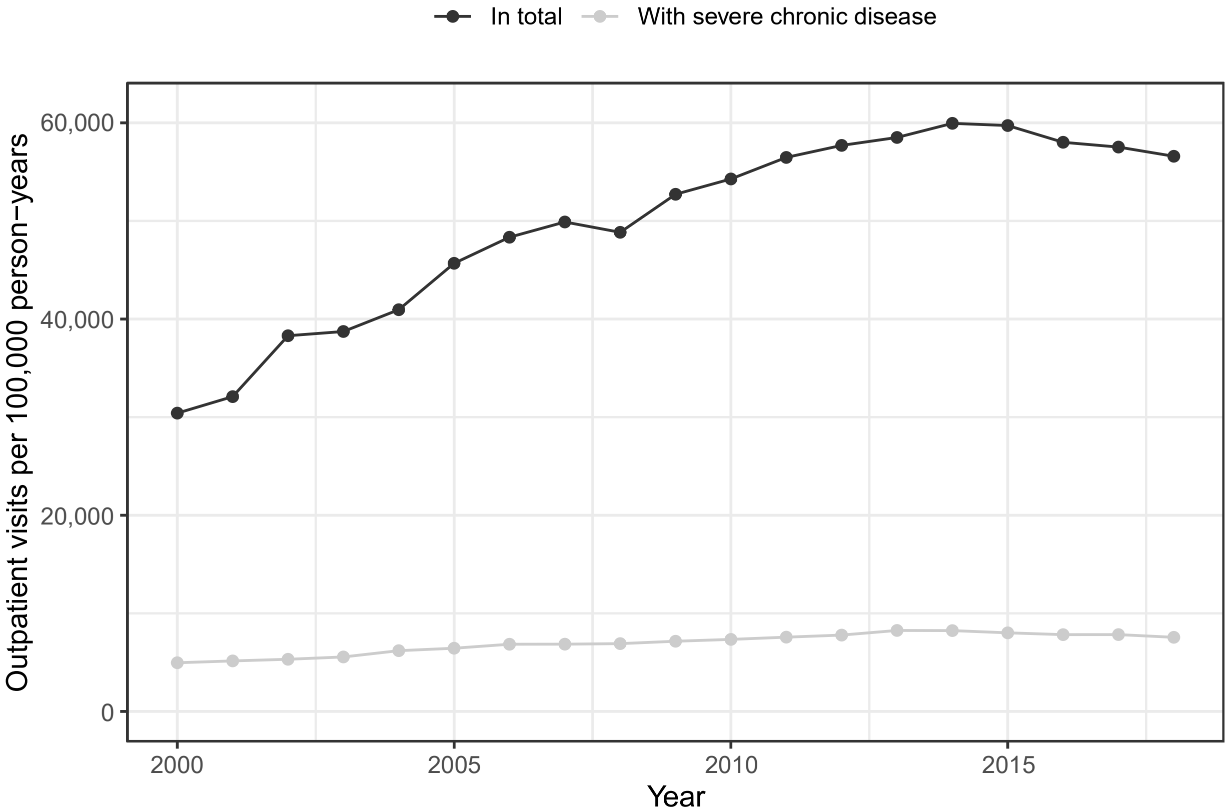

Supplementary Figure S12. Incidence rate of outpatient visits at public somatic hospitals, i.e., tertiary and secondary hospitals combined, by year. The included outpatient records starting prior to 2000 without visit dates are not shown. See Supplementary Table S5 for the person-time

Supplementary Discussion: Changes Over Time and Administrative Changes

Figures 3 and 4 show remarkable changes in the number of in- and outpatient contacts with the tertiary hospitals throughout the study period 2000-2018. Many of the drastic changes seen between adjacent years might be due to administrative changes. The fluctuations following structural changes were often lagged, presumably due to administrative coding practices moving at a slower pace than practical changes.

For admissions at Rigshospitalet, an upward trend started in 2009, flattened out around 2012, and was followed by a dramatic decrease starting in 2017 (Figure 3). The upward trend was initiated around two years after the introduction of the Regions in 2007. The decrease in 2017 might be explained by the introduction of a new electronic journaling system EPIC (Danish: Sundhedsplatform) in late 2016. At the same time, coding practices were changed, moving much of the responsibility for registrations from medical secretaries to physicians. We hypothesize that this may have resulting in less thorough recording of information. This could include, e.g., less diligent recording of infusions as admissions instead of outpatient visits. The change of journaling system in the Capital Region has been noted to have caused an actual reduction in hospital activity along with issues with registering the completed activities.[20] The relative scope of the two issues is unknown. A sizable decrease in both admissions and number of unique patients admitted for hospitalization at Rigshospitalet was observed from 2016 to 2017 (Figure 3 and Supplementary Figure S20). However, the number of days spent in hospital did not drop anywhere near as much (Supplementary Figure S17). This could be explained by a move towards more outpatient visits in place of admissions of a

short duration. The decrease also coincided with the fusion of Rigshospitalet with Glostrup Hospital, which is not included prior to the fusion.

The changes seen over time at Odense University Hospital included a decrease in admissions in 2014 (Figure 3), and a decrease in outpatient visit in 2016 (Figure 4). Notably, these changes did not line up with the opening of Hans Christian Andersen Children's Hospital at Odense University Hospital in 2008. It would thus seem that not all organizational changes have a noticeable impact on hospital contacts. Both Odense University Hospital and Aarhus University Hospital saw a slight decline in the number of admissions towards the later part of the study period 2000-2018 (Figure 3), which could be related to the centralization of treatment for congenital heart disease at Rigshospitalet in 2016.

Aarhus University Hospital underwent many structural changes during 2000-2018. In 2004, Aarhus County Hospital (Danish: Aarhus Amtssygehus) was merged with Aarhus Hospital (Danish: Aarhus Sygehus). Contacts with Aarhus County Hospital were not included in the study prior to the merger with Aarhus Hospital. However, Aarhus Hospital was included. This local restructuring could explain the decrease in admissions seen at Aarhus University Hospital in the mid-2000s (Figure 3). In 2011, Aarhus University Hospital was formally established by merging Skejby University Hospital with Aarhus Hospital, providing a possible explanation for the decrease in outpatient visits observed in the early 2010s (Figure 4).

Compared to the other hospitals, Aalborg University Hospital experienced relatively few drastic changes over the study period. Despite this, the hospital did experience several organizational changes during the years 2000-2018. Indeed, it was not associated with a university until in 2003 when a partnership with Aarhus University Hospital was initiated. In 2013, Aalborg University Hospital assumed its current name when a collaboration with Aalborg University was established. Perhaps the most notable development over time at Aalborg University Hospital is that the number of contacts (in particular outpatient visits) with a severe chronic disease did not follow suit with the overall increase in contacts.

Supplementary Tables – Contacts at Secondary Hospitals

Supplementary Table S9. Admissions and outpatient visits at secondary hospitals in Denmark. Male patients, severe chronic disease, and region are provided in contacts. Age is median (interquartile range) age in years at the start of the contact.

**Admissions**	1,971,559
Unique patients	926,410
Days in hospital<br>With severe chronic disease	6,271,560<br>758,177 (12.09%)
Male patients	1,066,477 (54.09%)
Age	2.81 (0.38-10.04)
Severe chronic disease	166,886 (8.46%)
Patients from the hospital’s region	1,884,633 (95.59%)
**Outpatient visits**	7,322,829
Unique patients	1,453,826
Male patients	3,763,266 (51.39%)
Age	9.17 (2.82-14.05)
Severe chronic disease	743,911 (10.16%)
Patients from the hospital’s region	7,001,777 (95.62%)

Supplementary Table S10. The ten most common non-birth main diagnoses (3-digit ICD-10 code) for admissions at secondary hospitals. Percentages calculated relative to the total number of admissions.

	**ICD-10**		**n (%)**
**For the secondary hospitals combined**			
1	Z03	Medical observation for suspected disease/conditions, ruled out	172,289 (8.74%)
2	R10	Abdominal and pelvic pain	83,008 (4.21%)
3	J45	Asthma	57,935 (2.94%)
4	R56	Convulsions, not elsewhere classified	52,219 (2.65%)
5	J20	Acute bronchitis	51,124 (2.59%)
6	B34	Viral infection of unspecified site	47,629 (2.42%)
7	A09	Infectious gastroenteritis and colitis, unspecified	43,763 (2.22%)
8	Z76	Encountering health services in other circumstances	40,611 (2.06%)
9	J05	Acute obstructive laryngitis [croup] and epiglottitis	40,191 (2.04%)
10	J35	Chronic diseases of tonsils and adenoids	40,041 (2.03%)

Supplementary Table S11. The ten most common main diagnoses (3-digit ICD-10 code) for outpatient visits at secondary hospitals. Percentages calculated relative to the total number of outpatient visits.

	**ICD-10**		**n (%)**
**For the secondary hospitals combined**			
1	Z01	Other special examinations and investigations of persons without complaint	1,081,095 (14.76%)
2	Z13	Special screening examinations for other diseases and disorders	494,283 (6.75%)
3	Z03	Medical observation for suspected diseases/conditions, ruled out	473,350 (6.46%)
4	J45	Asthma	337,127 (4.60%)
5	Z09	Follow-up examination after treatment for conditions other than malignant neoplasms	230,721 (3.15%)
6	E10	Type 1 diabetes mellitus	187,940 (2.57%)
7	Z50	Care involving use of rehabilitation procedures	116,028 (1.58%)
8	R62	Lack of expected normal physiological development in childhood and adults	115,618 (1.58%)
9	S52	Fracture of forearm	113,176 (1.55%)
10	R32	Unspecified urinary incontinence	97,392 (1.33%)

Supplementary Figures and Tables – Inpatients at Tertiary Hospitals

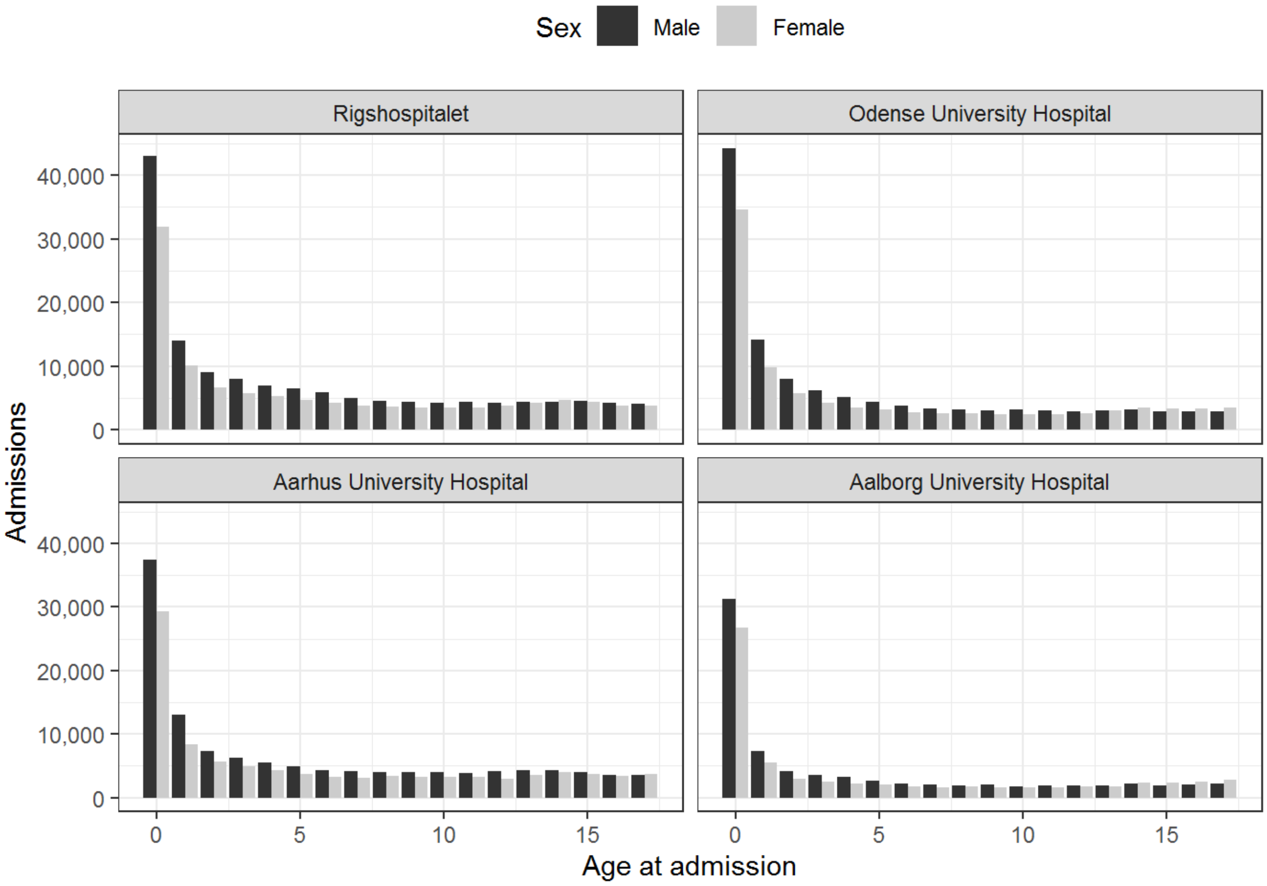

Supplementary Figure S13. Admissions by age at admission and sex

Supplementary Table S12. Sex of patients admitted with a diagnosis of a severe chronic disease. Percentages relative to admissions with severe chronic disease at the hospital

Hospital	Male	Female
Rigshospitalet	58,237 (56.9%)	44,057 (43.1%)
Odense University Hospital	22,969 (55.9%)	18,102 (44.1%)
Aarhus University Hospital	38,961 (55.4%)	31,349 (44.6%)
Aalborg University Hospital	11,564 (56.3%)	8,973 (43.7%)

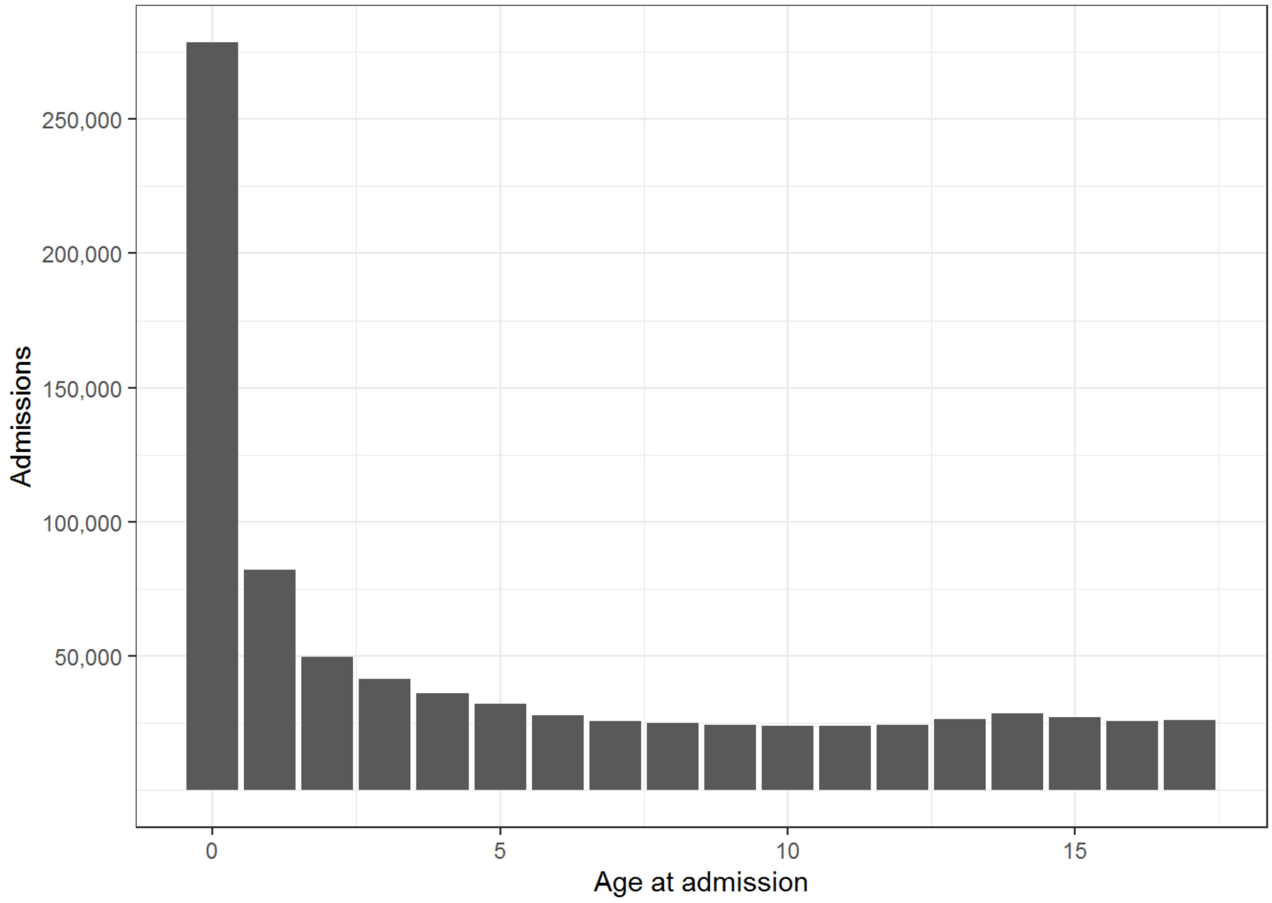

Supplementary Figure S14. Admissions by age

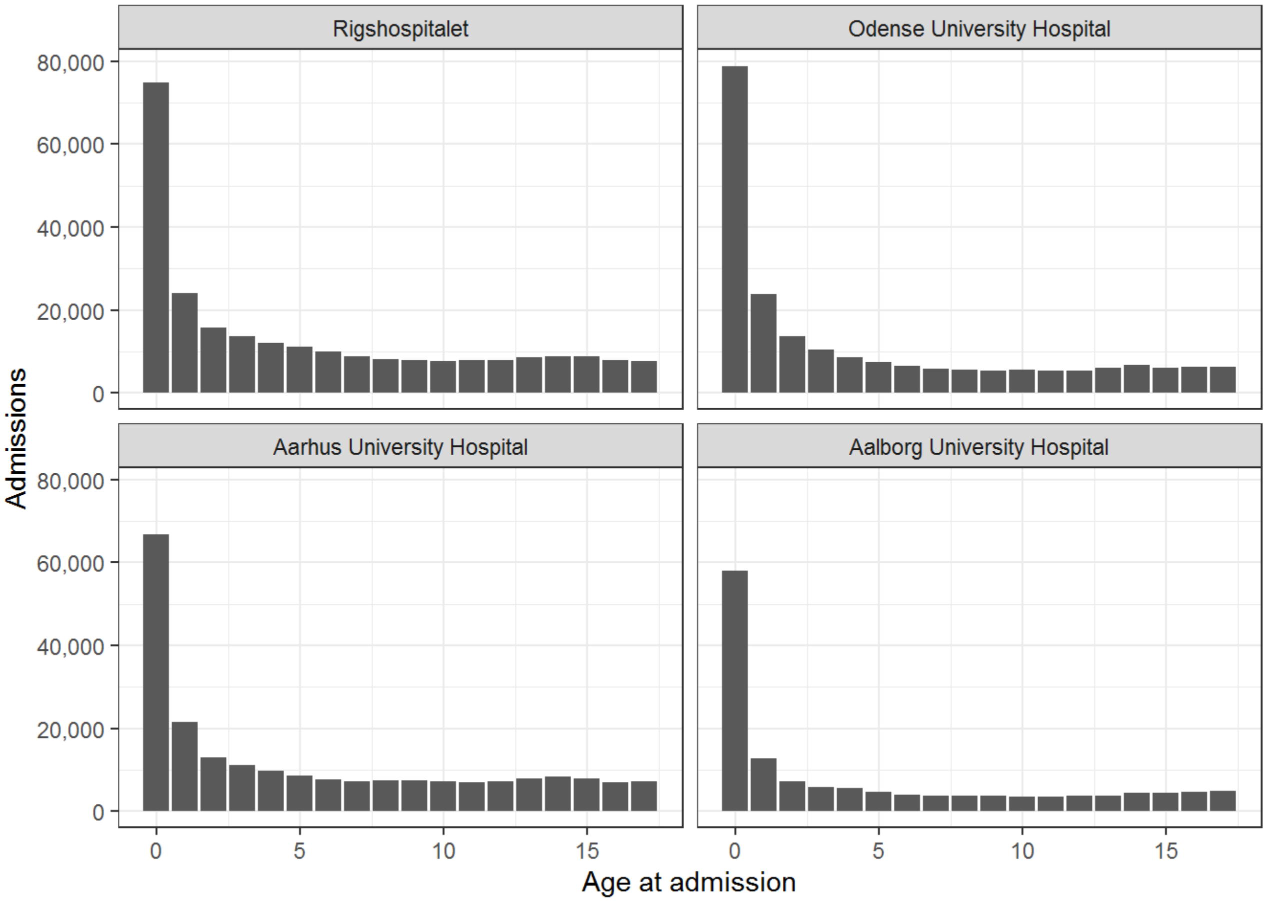

Supplementary Figure S15. Admissions by age and hospital

Supplementary Table S13. Admissions by year. Admissions starting in 1999 are not shown

Admission	Rigshospitalet	Odense University Hospital	Aarhus University Hospital	Aalborg University Hospital
**2000**	8,608	9,476	11,378	6,316
**2001**	8,843	10,441	12,221	6,555
**2002**	8,575	10,885	12,957	6,348
**2003**	9,104	10,874	13,333	6,302
**2004**	9,679	11,488	13,659	6,141
**2005**	10,385	12,526	14,923	7,298
**2006**	11,237	12,898	10,552	7,999
**2007**	10,943	13,311	10,229	8,039
**2008**	10,258	13,047	9,725	7,757
**2009**	12,291	13,508	10,250	8,826
**2010**	15,757	13,451	11,623	8,439
**2011**	18,430	13,030	11,559	9,341
**2012**	19,525	12,704	11,815	9,646
**2013**	19,758	13,295	12,076	9,156
**2014**	19,363	9,990	11,385	8,455
**2015**	18,295	8,898	10,819	6,753
**2016**	18,006	8,224	11,144	6,455
**2017**	11,349	8,463	10,930	6,190
**2018**	11,663	7,925	9,727	6,471

Supplementary Table S14. Admissions with severe chronic disease by year of admission. Admissions starting in 1999 are not shown

Admission	Rigshospitalet	Odense University Hospital	Aarhus University Hospital	Aalborg University Hospital
**2000**	3,815	1,787	4,056	626
**2001**	3,951	2,101	4,183	900
**2002**	3,500	2,296	5,009	923
**2003**	3,913	2,061	5,002	939
**2004**	4,656	2,538	5,256	945
**2005**	5,204	2,338	5,449	1,000
**2006**	5,656	2,536	3,331	1,216
**2007**	5,147	2,376	2,913	1,271
**2008**	4,971	2,439	2,999	1,349
**2009**	5,468	2,533	2,940	1,325
**2010**	6,623	2,497	3,429	1,286
**2011**	6,704	2,270	3,351	1,318
**2012**	6,899	2,286	3,609	1,445
**2013**	7,066	2,220	3,547	1,418
**2014**	6,955	1,830	3,266	1,258
**2015**	7,106	1,778	2,926	908
**2016**	6,588	1,751	3,170	807
**2017**	3,804	1,766	3,079	721
**2018**	4,232	1,641	2,782	875

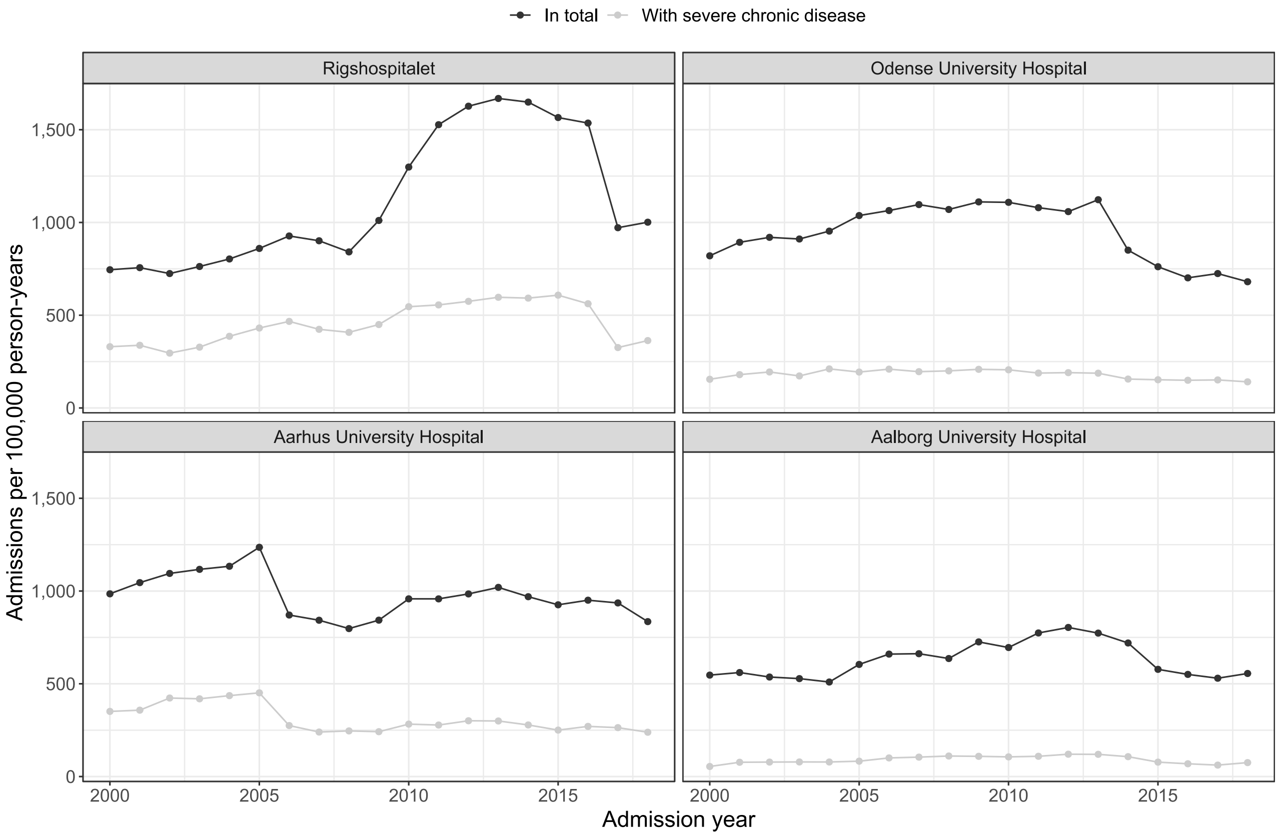

Supplementary Figure S16. Incidence rate of admissions at the four tertiary hospitals relative to the population of children and adolescents 0-17 years old living in Denmark. See Supplementary Table S5 for the person-time

Supplementary Table S15. The ten most common diagnoses of a severe chronic disease (3-digit ICD-10 code) for admissions. Note that the severe chronic disease is not necessarily the main diagnosis for the admission, and thus admissions can have more than one such diagnosis attached. Percentages are relative to the total number of admissions with a severe chronic disease

	**ICD-10**		**n (%)**
**For the hospitals combined**			
1	C91	Lymphoid leukemia	37,641 (16.07)
2	G40	Epilepsy	13,407 (5.72)
3	Q21	Congenital malformations of cardiac septa	12,138 (5.18)
4	Q62	Congenital obstructive defects of renal pelvis and congenital malformations of ureter	9,168 (3.91)
5	C71	Malignant neoplasm of brain	8,179 (3.49)
6	G80	Cerebral palsy	7,183 (3.07)
7	Q25	Congenital malformations of great arteries	7,103 (3.03)
8	Q37	Cleft palate with cleft lip	5,276 (2.25)
9	Q43	Other congenital malformations of intestine	4,973 (2.12)
10	G91	Hydrocephalus	4,964 (2.12)
**For Rigshospitalet**			
1	C91	Lymphoid leukemia	16,371 (16.00)
2	Q21	Congenital malformations of cardiac septa	4,763 (4.66)
3	Q37	Cleft palate with cleft lip	4,240 (4.14)
4	G40	Epilepsy	4,112 (4.02)
5	C71	Malignant neoplasm of brain	3,694 (3.61)
6	Q62	Congenital obstructive defects of renal pelvis and congenital malformations of ureter	3,612 (3.53)
7	Q25	Congenital malformations of great arteries	2,767 (2.70)
8	G80	Cerebral palsy	2,618 (2.56)
9	E84	Cystic fibrosis	2,612 (2.55)
10	G91	Hydrocephalus	2,552 (2.49)
**For Odense University Hospital**			
1	C91	Lymphoid leukemia	4,566 (11.12)
2	G40	Epilepsy	3,710 (9.03)
3	Q21	Congenital malformations of cardiac septa	2,784 (6.78)
4	Q43	Other congenital malformations of intestine	2,471 (6.02)
5	K21	Gastro-esophageal reflux disease	2,284 (5.56)
6	E10	Type 1 diabetes mellitus	1,572 (3.83)
7	G80	Cerebral palsy	1,501 (3.65)
8	Q25	Congenital malformations of great arteries	1,423 (3.46)
9	C71	Malignant neoplasm of brain	1,353 (3.29)
10	Q39	Congenital malformations of esophagus	1,239 (3.02)
**For Aarhus University Hospital**			
1	C91	Lymphoid leukemia	12,430 (17.68)
2	Q62	Congenital obstructive defects of renal pelvis and congenital malformations of ureter	4,645 (6.61)
3	Q21	Congenital malformations of cardiac septa	3,695 (5.26)
4	G40	Epilepsy	2,835 (4.03)
5	C40	Malignant neoplasm of bone and articular cartilage of limbs	2,662 (3.79)
6	Q25	Congenital malformations of great arteries	2,468 (3.51)
7	C71	Malignant neoplasm of brain	2,320 (3.30)
8	G80	Cerebral palsy	2,098 (2.98)
9	Q05	Spina bifida	1,656 (2.36)
10	Q64	Other congenital malformations of urinary system	1,549 (2.20)
**For Aalborg University Hospital**			
1	C91	Lymphoid leukemia	4,274 (20.81)
2	G40	Epilepsy	2,750 (13.39)
3	E10	Type 1 diabetes mellitus	1,329 (6.47)
4	G80	Cerebral palsy	966 (4.70)
5	Q21	Congenital malformations of cardiac septa	896 (4.36)
6	C71	Malignant neoplasm of brain	812 (3.95)
7	K50	Crohn disease	691 (3.36)
8	G82	Paraplegia and tetraplegia	565 (2.75)
9	G91	Hydrocephalus	536 (2.61)
10	Q25	Congenital malformations of great arteries	445 (2.17)

Supplementary Table S16. Most common secondary diagnoses for admissions with the two most common medical observation main diagnoses (ICD-10: Z03 and Z76). Most outpatient visits with these main diagnoses did not have secondary diagnoses. Percentages are relative to the number of outpatient visits with the main diagnosis. The percentages of outpatient visits that had a secondary diagnosis was 16,563 (30.3%) for main diagnosis Z03, and 6,146 (54.2%) for main diagnosis Z76.

	**ICD-10**		**n (%)**
**For main diagnosis Z03 (Medical observation for suspected diseases/conditions, ruled out)**			
1	P70	Transitory disorders of carbohydrate metabolism specific to newborn	1,303 (2.39)
2	Z03	Encounter for medical observation for suspected diseases and conditions ruled out	819 (1.50)
3	Z13	Encounter for screening for other diseases and disorders	695 (1.27)
4	B34	Viral infection of unspecified site	591 (1.08)
5	P03	Newborn (suspected to be) affected by other complications of labor and delivery	505 (0.92)
**For main diagnosis Z76 (Encountering health services in other circumstances)**			
1	Z13	Encounter for screening for other diseases and disorders	1,605 (14.15)
2	Z03	Encounter for medical observation for suspected diseases and conditions ruled out	65 (0.57)
3	P07	Disorders of newborn related to short gestation and low birth weight, not elsewhere classified	61 (0.54)
4	P01	Newborn (suspected to be) affected by maternal complications of pregnancy	52 (0.46)
5	P22	Respiratory distress of newborn	34 (0.30)

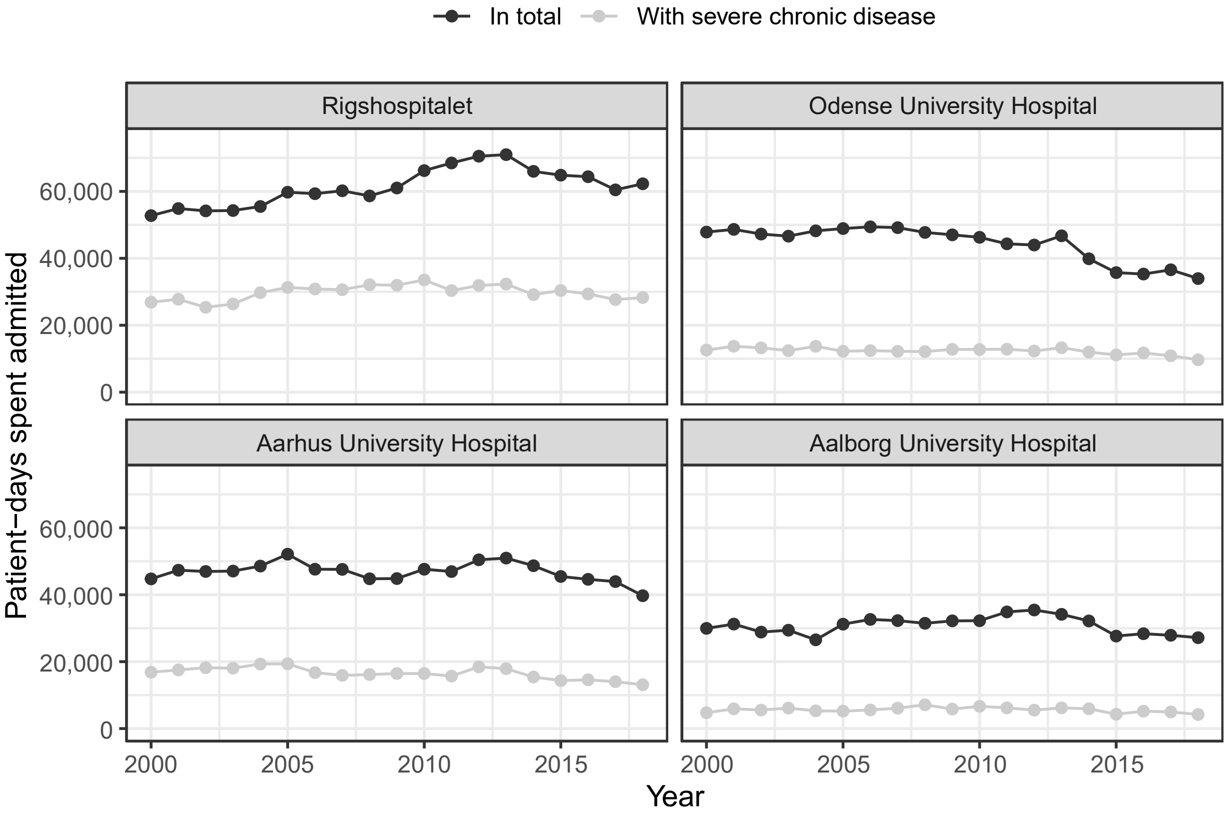

Supplementary Figure S17. Admission days per year and hospital. An admission with same day discharge is counted as lasting one day. Note that if a patient is admitted to a tertiary hospital the same day they are discharged from another, this day is counted as a day spent in hospital at both

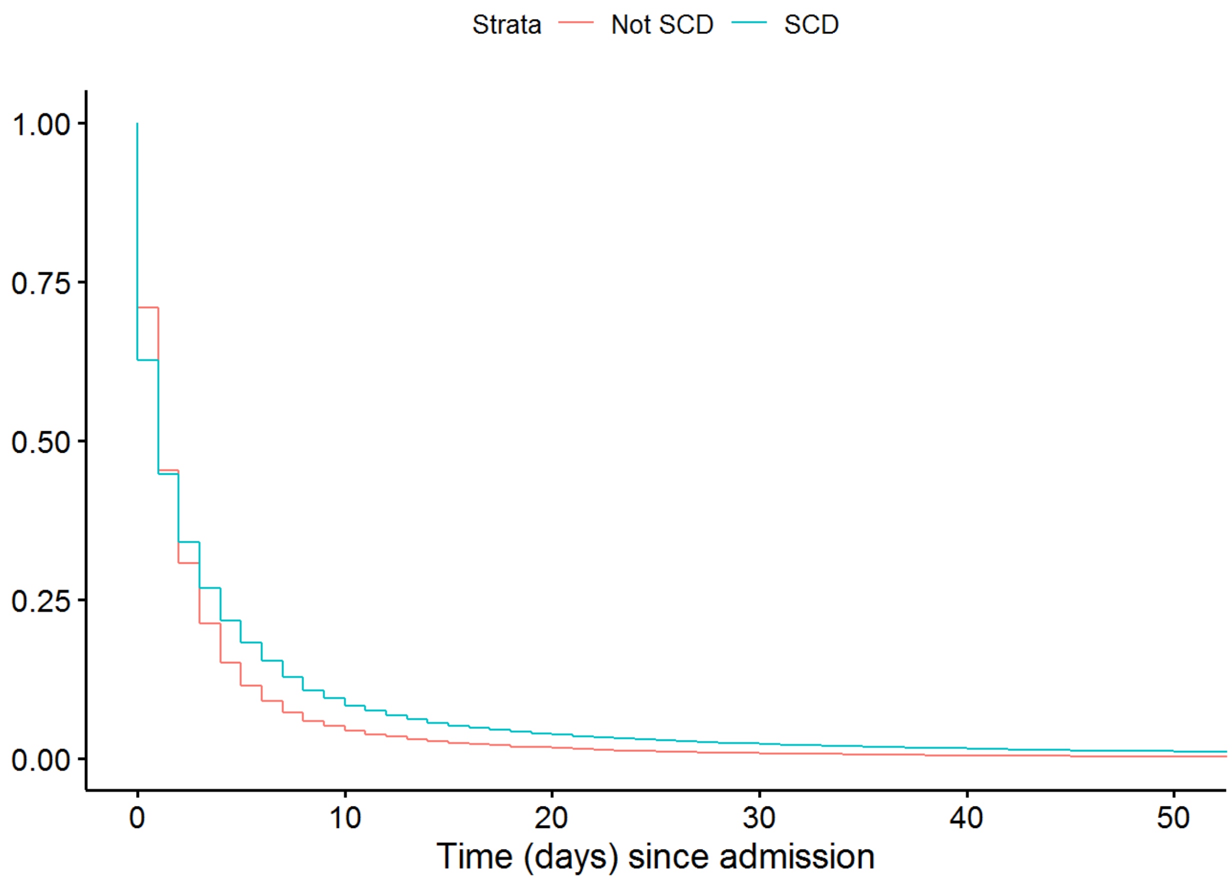

Supplementary Figure S18. Estimated survival function (Kaplan-Meier estimate) of time from admission to discharge (in days) for admissions with and without a diagnosis of a severe chronic disease (SCD)

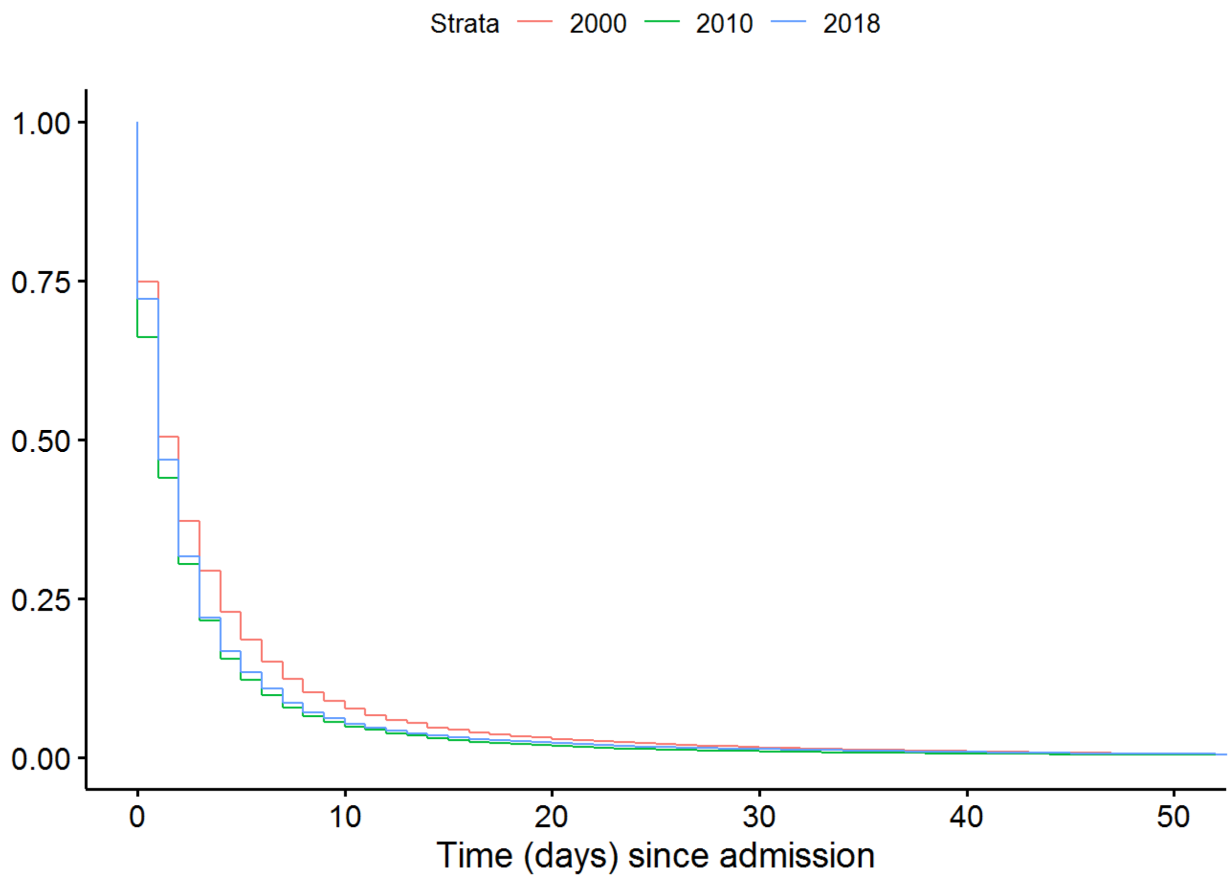

Supplementary Figure S19. Estimated survival function (Kaplan-Meier estimate) of time from admission to discharge (in days) for admissions starting in the years 2000, 2010, and 2018

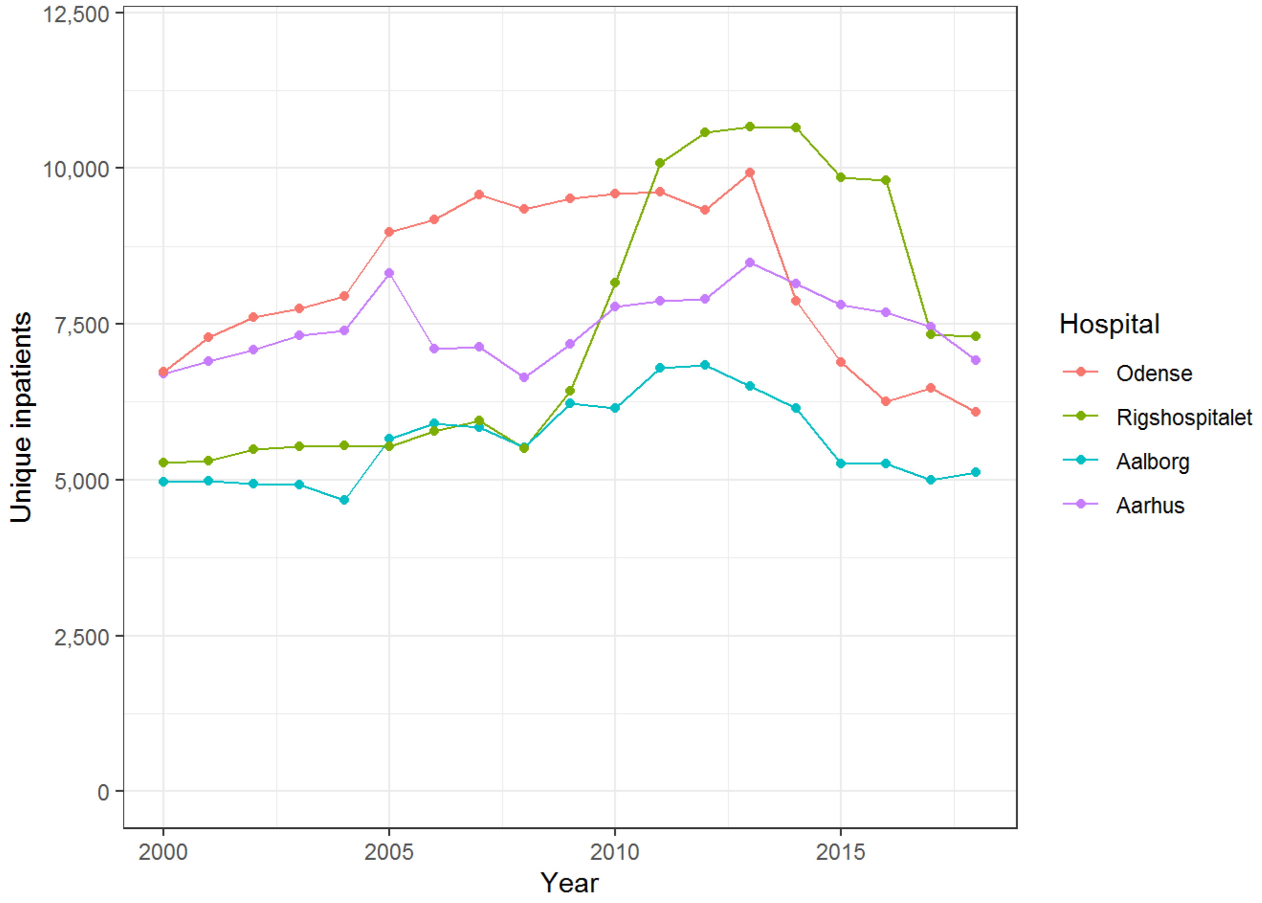

Supplementary Figure S20. Unique patients with an admission overlapping the year by hospital

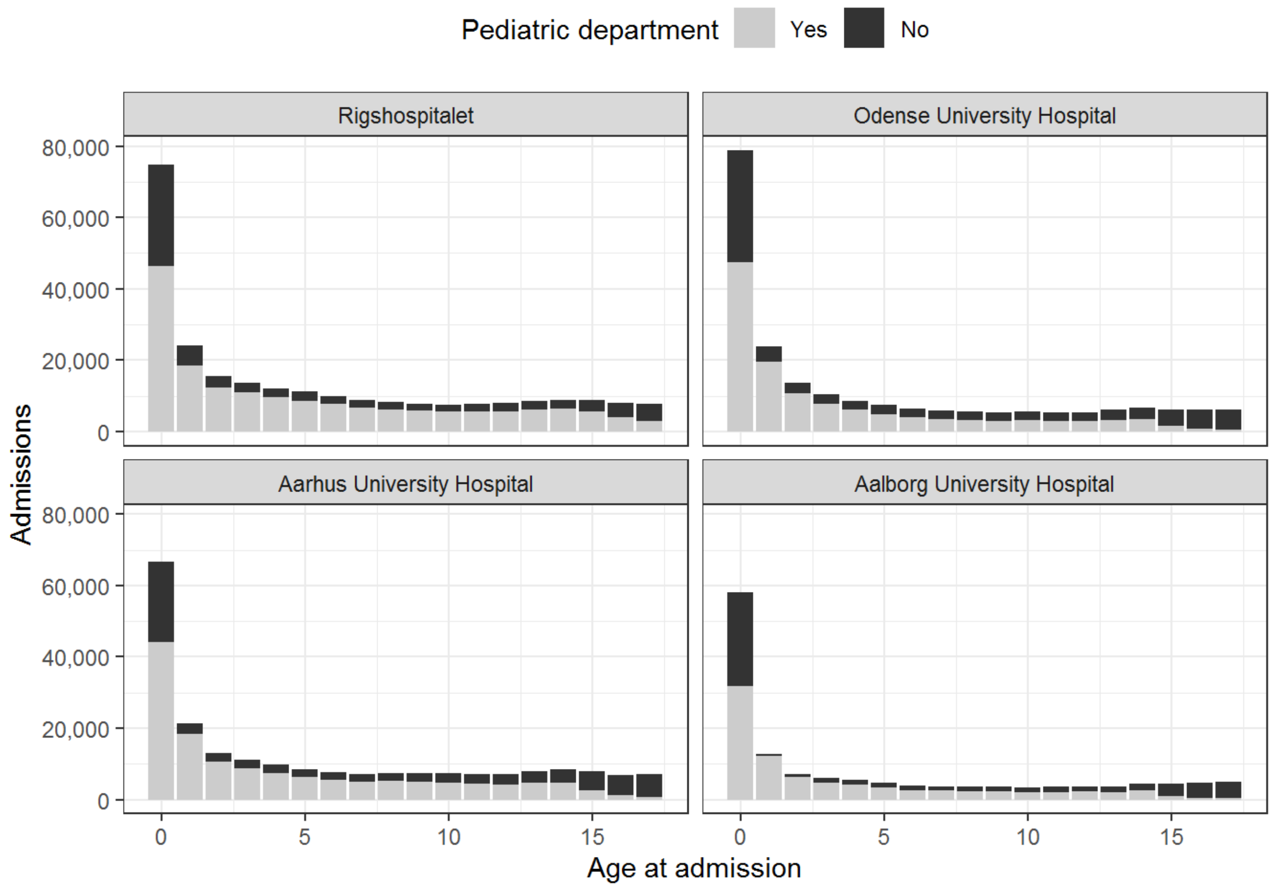

Supplementary Figure S21. Admissions by age at time of admission and whether the responsible department was pediatric

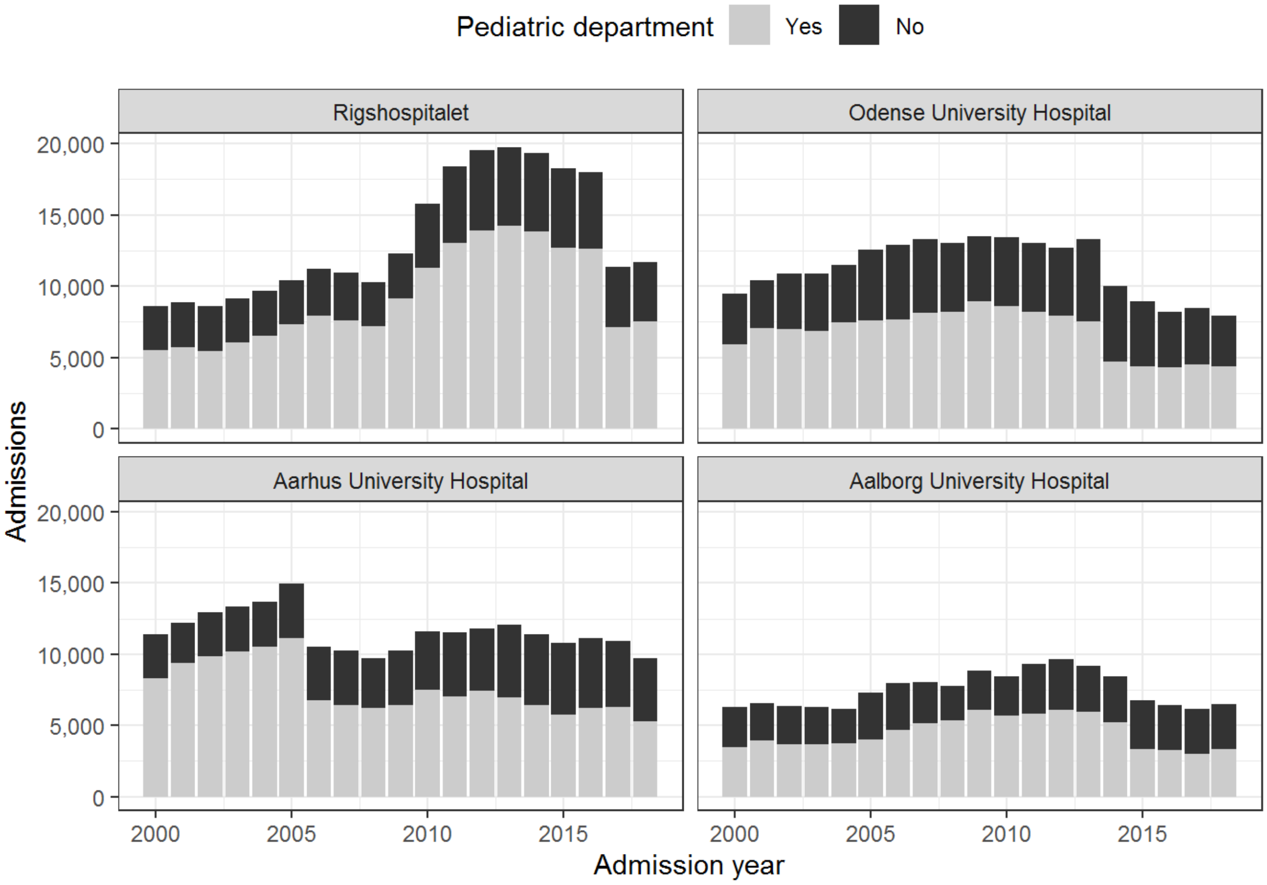

Supplementary Figure S22. Admissions by year of admission and whether the responsible department was pediatric

Supplementary Table S17. The patients' Region of residence at time of admission. The Region in which the hospital is placed is indicated in bold. Percentages are relative to the hospital

Region	Rigshospitalet	Odense University Hospital	Aarhus University Hospital	Aalborg University Hospital
Capital Region	**189,839 (75.3%)**	1,641 (0.8%)	2,080 (0.9%)	636 (0.4%)
Region Zealand	47,466 (18.8%)	2,990 (1.4%)	2,750 (1.2%)	238 (0.2%)
Region of Southern Denmark	7,102 (2.8%)	**198,949 (92.7%)**	18,928 (8.6%)	644 (0.5%)
Central Region Denmark	4,956 (2.0%)	7,627 (3.6%)	**184,109 (83.5%)**	1,916 (1.3%)
North Denmark Region	2,377 (0.9%)	2,559 (1.2%)	12,373 (5.6%)	**139,003 (97.5%)**
Missing	411 (0.2%)	763 (0.4%)	120 (0.1%)	85 (0.1%)

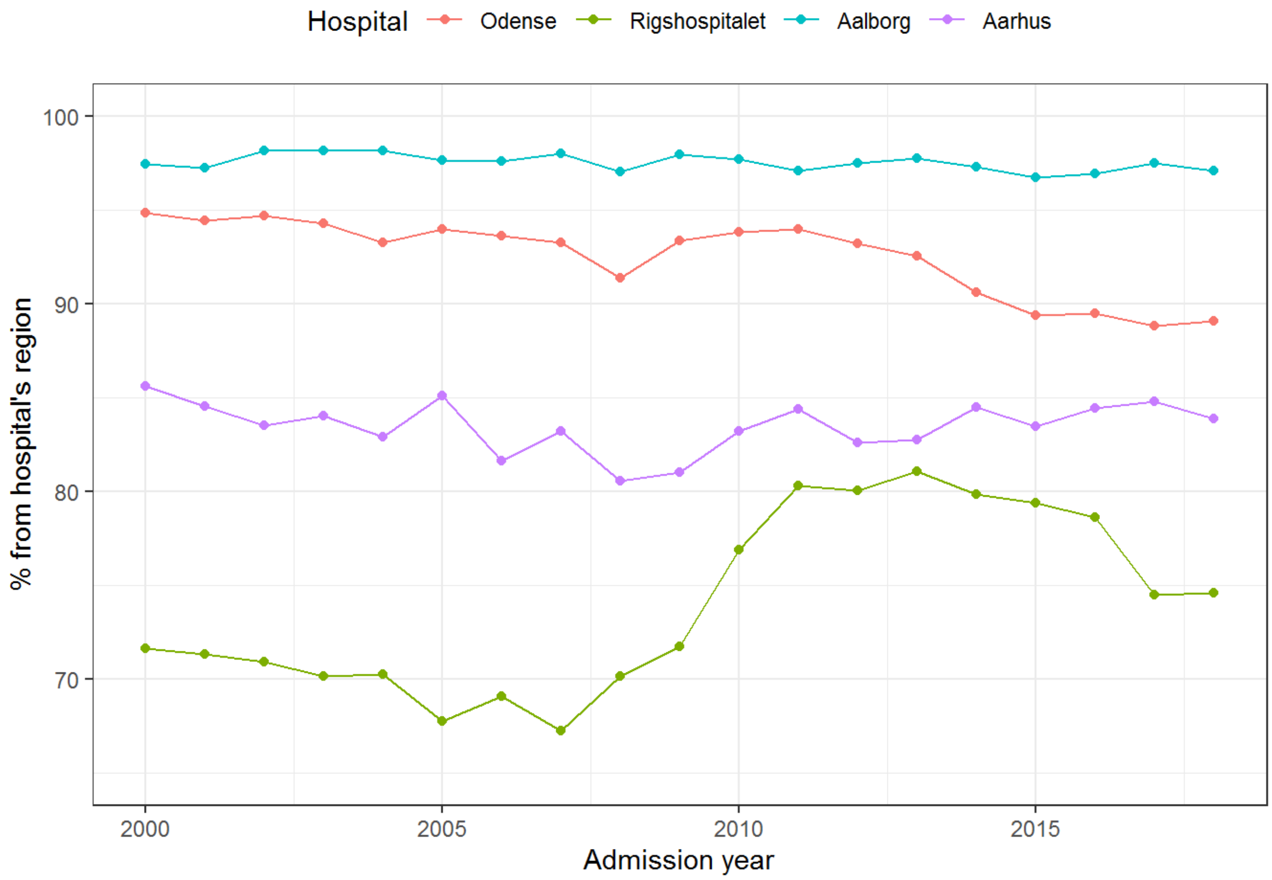

Supplementary Figure S23. Percent of admissions starting in 2000-2018, where the patient lived in the hospital's region at the time of admission.

Supplementary Figures and Tables – Outpatients at Tertiary Hospitals

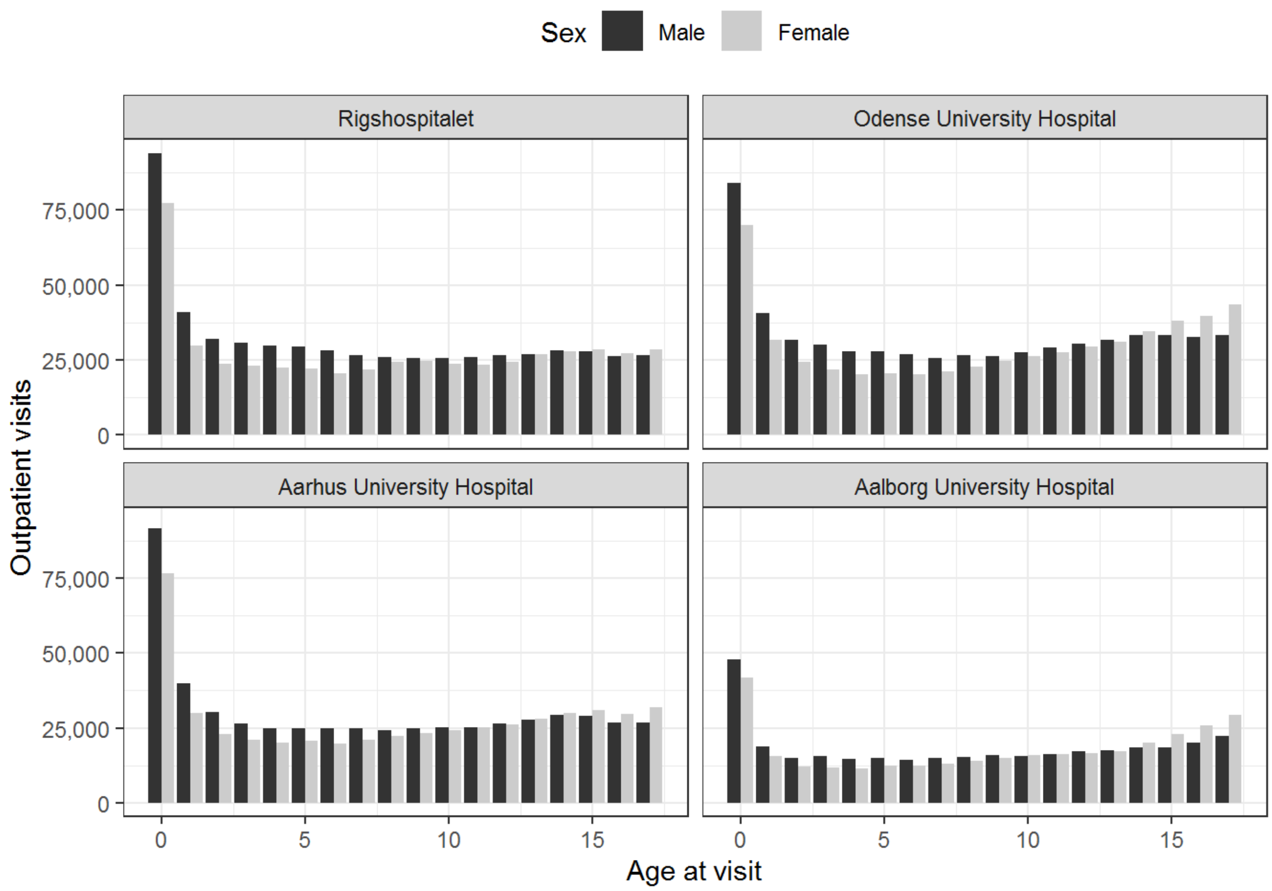

Supplementary Figure S24. Outpatient visits by age at time of visit grouped by sex at each of the four hospitals

Supplementary Table S18. Sex of patients for outpatient visits with a severe chronic disease. Percentages relative to outpatient visits with a severe chronic disease at the hospital

Hospital	Male	Female
Rigshospitalet	202,214 (55.2%)	163,831 (44.8%)
Odense University Hospital	103,273 (54.4%)	86,424 (45.6%)
Aarhus University Hospital	105,206 (53.3%)	92,353 (46.7%)
Aalborg University Hospital	39,223 (53.3%)	34,308 (46.7%)

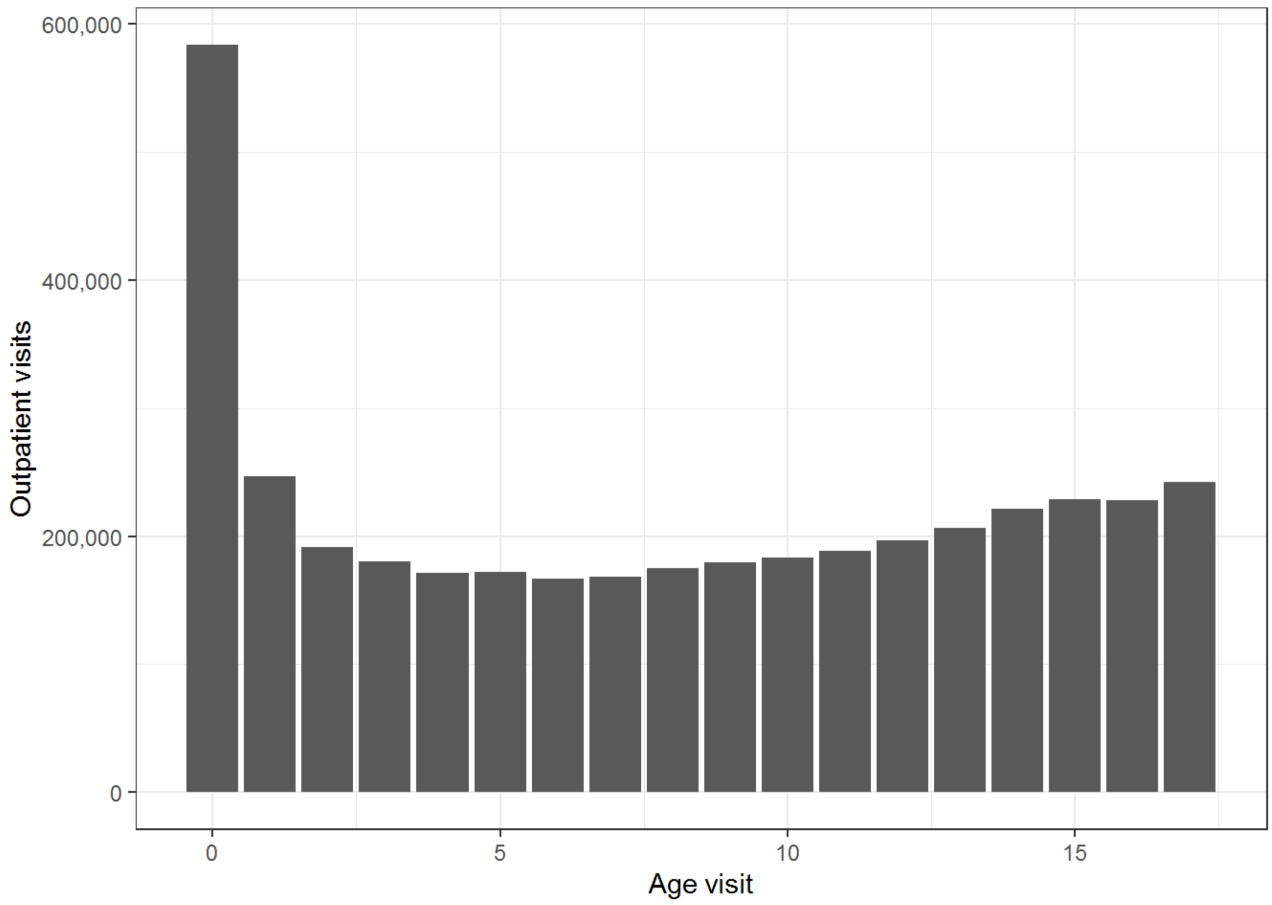

Supplementary Figure S25. Age at time of outpatient visits at the four tertiary hospitals combined

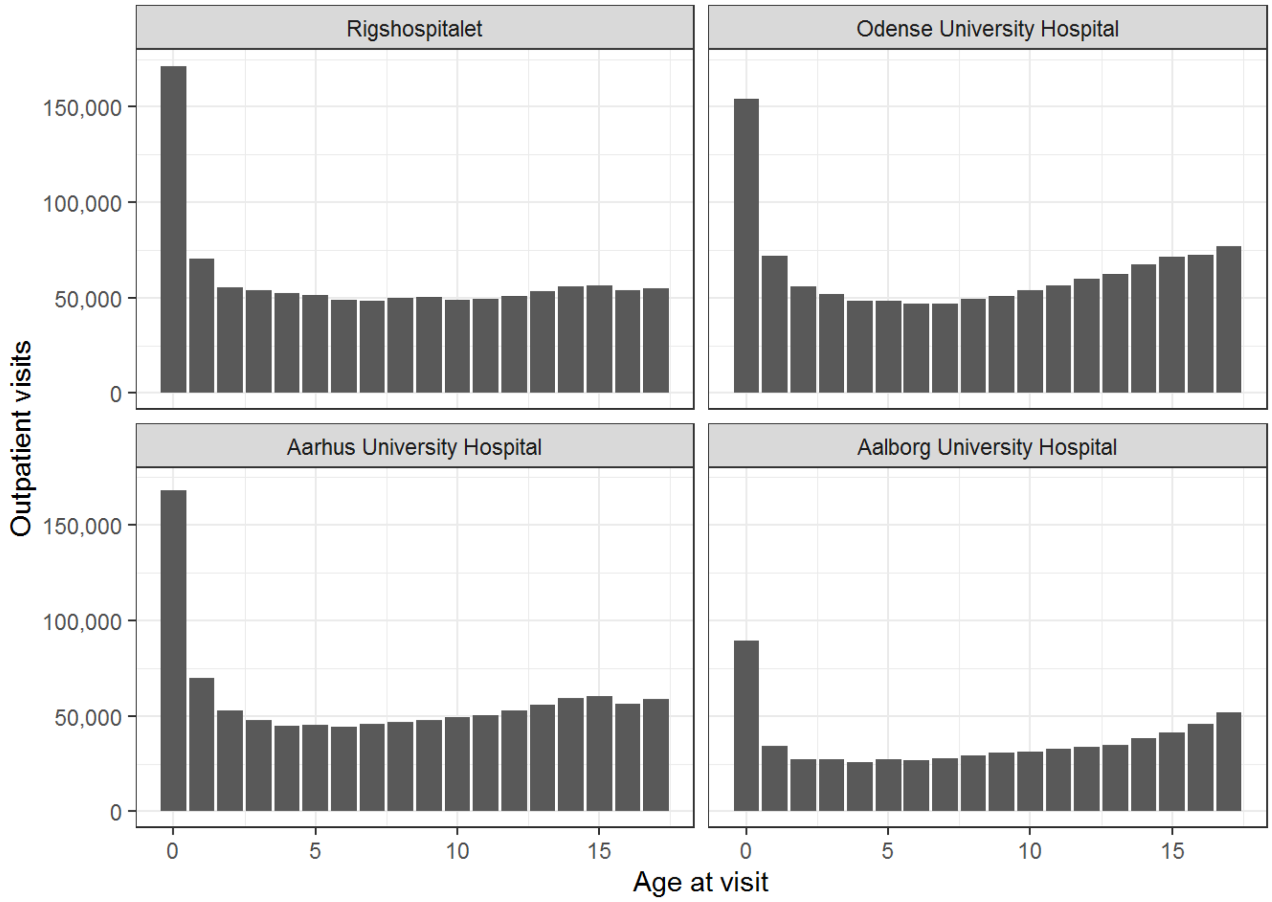

Supplementary Figure S26. Age at time of outpatient visits for the four tertiary hospitals

Supplementary Table S19. Outpatient visits per year. Records without visit dates starting before 2000 are not included

Admission	Rigshospitalet	Odense University Hospital	Aarhus University Hospital	Aalborg University Hospital
**2000**	35,660	27,684	28,914	17,740
**2001**	36,064	31,603	31,918	18,455
**2002**	37,231	39,099	34,292	22,940
**2003**	39,951	45,174	36,230	25,419
**2004**	44,292	48,757	37,109	25,182
**2005**	48,233	52,742	42,825	26,973
**2006**	51,469	54,048	53,731	29,038
**2007**	53,649	53,512	54,748	30,364
**2008**	53,411	56,121	50,833	30,922
**2009**	54,944	60,595	57,372	33,892
**2010**	57,420	63,195	64,246	31,489
**2011**	60,282	66,606	72,039	38,965
**2012**	61,442	70,233	78,400	40,785
**2013**	67,557	79,896	72,397	43,143
**2014**	66,257	87,804	72,851	46,016
**2015**	68,390	87,430	68,617	47,283
**2016**	74,529	73,411	69,217	48,717
**2017**	81,427	72,938	67,900	48,938
**2018**	82,947	73,679	62,829	49,755

Supplementary Table S20. Outpatient visits with severe chronic disease by year. Records without visit dates starting before 2000 are not included

Admission	Rigshospitalet	Odense University Hospital	Aarhus University Hospital	Aalborg University Hospital
**2000**	13,982	5,644	6,620	3,210
**2001**	14,504	6,139	7,013	3,274
**2002**	13,803	6,476	7,072	3,519
**2003**	15,373	6,651	7,376	3,598
**2004**	17,363	7,693	7,878	3,755
**2005**	18,393	7,861	8,602	3,465
**2006**	18,671	8,621	11,407	3,521
**2007**	17,102	8,968	11,260	3,293
**2008**	17,968	9,748	10,979	3,336
**2009**	19,087	10,314	11,547	3,497
**2010**	20,225	10,975	12,005	3,443
**2011**	20,637	11,491	12,259	3,591
**2012**	20,382	12,046	12,997	3,671
**2013**	23,924	12,995	12,806	4,267
**2014**	23,772	13,392	12,108	4,671
**2015**	22,051	13,362	11,081	4,824
**2016**	22,945	12,410	11,486	5,036
**2017**	22,429	12,809	11,674	4,830
**2018**	23,429	12,092	11,310	4,724

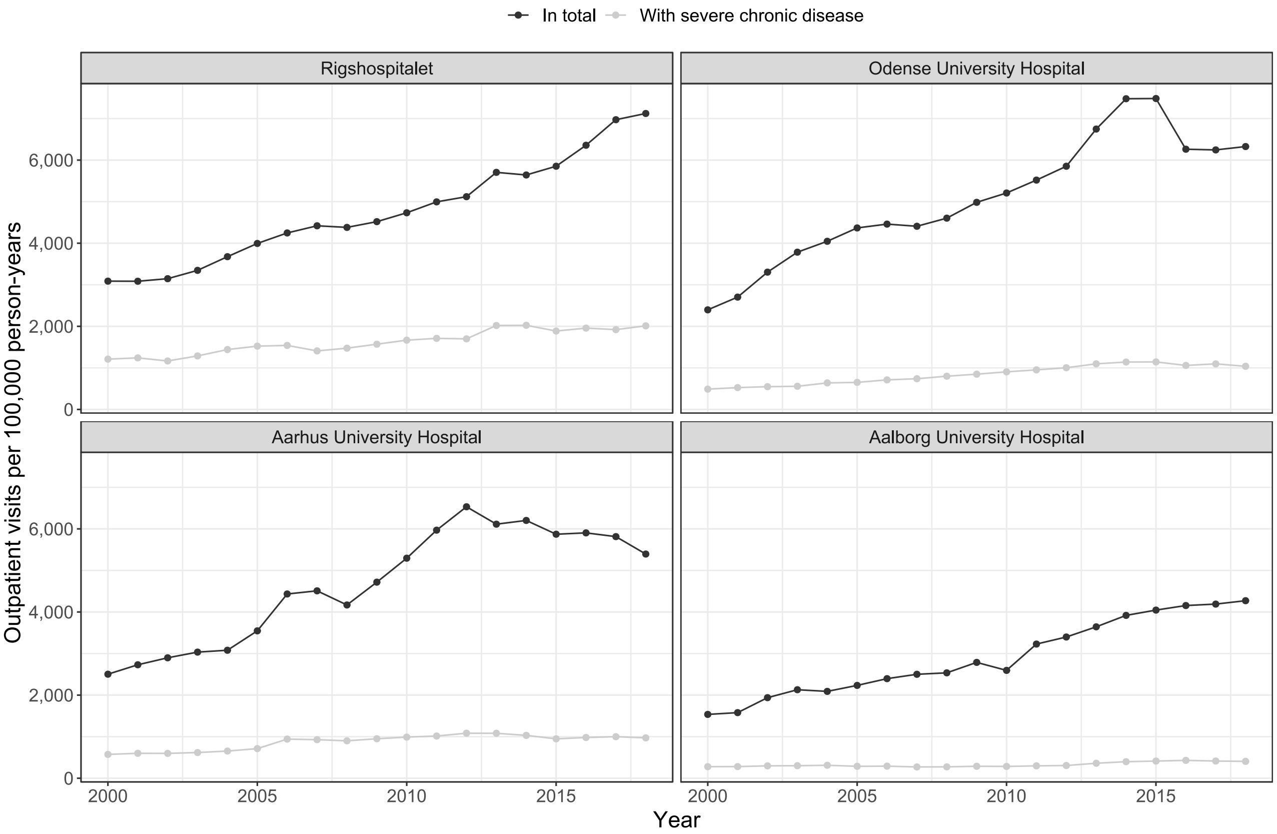

Supplementary Table S27. Incidence rate of outpatient visits at the four tertiary hospitals relative to the population of children and adolescents 0-17 years old living in Denmark. See Supplementary Table S5 for the person-time

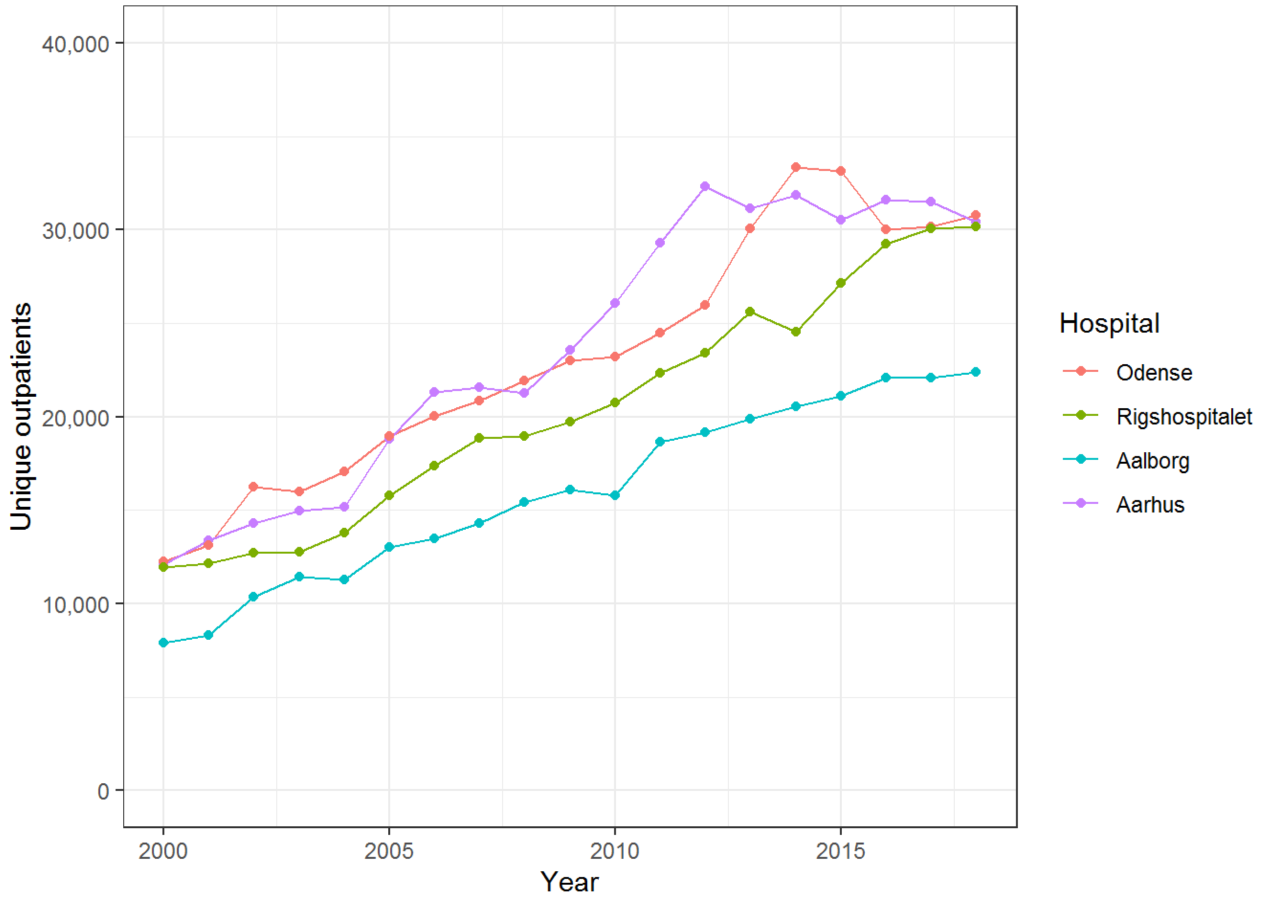

Supplementary Figure S28. Unique outpatients seen for at least one outpatient visit for the years 2000-2018

Supplementary Table S21. Top 10 most common diagnoses of severe chronic diseases (3-digit ICD-10 code) for outpatient visits. Note that the severe chronic diagnosis is not necessarily the main diagnosis for the visits, and thus visits can have more than one such diagnosis attached. Percentages are relative to the total number of outpatient visits with a severe chronic disease

	**ICD-10**		**n (%)**
**For the hospitals combined**			
1	C91	Lymphoid leukemia	79,233 (9.58)
2	Q21	Congenital malformations of cardiac septa	51,360 (6.21)
3	E10	Type 1 diabetes mellitus	49,733 (6.01)
4	G40	Epilepsy	44,174 (5.34)
5	E84	Cystic fibrosis	43,344 (5.24)
6	G80	Cerebral palsy	38,506 (4.66)
7	Q87	Other specified congenital malformation syndromes affection multiple systems	28,091 (3.40)
8	N18	Chronic kidney disease	26,152 (3.16)
9	Q25	Congenital malformations of great arteries	21,242 (2.57)
10	C71	Malignant neoplasm of brain	21,227 (2.57)
**For Rigshospitalet**			
1	C91	Lymphoid leukemia	43,637 (11.92)
2	E84	Cystic fibrosis	25,542 (6.98)
3	Q21	Congenital malformations of cardiac septa	20,097 (5.49)
4	Q37	Cleft palate with cleft lip	16,510 (4.51)
5	Q87	Other specified congenital malformation syndromes affection multiple systems	12,502 (3.42)
6	G40	Epilepsy	11,777 (3.22)
7	C71	Malignant neoplasm of brain	11,187 (3.06)
8	G80	Cerebral palsy	10,245 (2.80)
9	Q25	Congenital malformations of great arteries	9,433 (2.58)
10	N18	Chronic kidney disease	8,784 (2.40)
**For Odense University Hospital**			
1	E10	Type 1 diabetes mellitus	21,124 (11.14)
2	C91	Lymphoid leukemia	15,834 (8.35)
3	G40	Epilepsy	14,084 (7.42)
4	G80	Cerebral palsy	12,919 (6.81)
5	Q21	Congenital malformations of cardiac septa	11,829 (6.24)
6	N18	Chronic kidney disease	11,755 (6.20)
7	K21	Gastro-esophageal reflux disease	6,338 (3.34)
8	Q87	Other specified congenital malformation syndromes affection multiple systems	5,560 (2.93)
9	G82	Paraplegia and tetraplegia	5,295 (2.79)
10	K50	Crohn disease	5,014 (2.64)
**For Aarhus University Hospital**			
1	E10	Type 1 diabetes mellitus	17,088 (8.65)
2	E84	Cystic fibrosis	16,873 (8.54)
3	Q21	Congenital malformations of cardiac septa	12,971 (6.57)
4	C91	Lymphoid leukemia	12,270 (6.21)
5	G40	Epilepsy	10,597 (5.36)
6	G80	Cerebral palsy	9,072 (4.59)
7	Q87	Other specified congenital malformation syndromes affection multiple systems	8,164 (4.13)
8	Q62	Congenital obstructive defects of renal pelvis and congenital malformations of ureter	6,370 (3.22)
9	Q05	Spina bifida	6,223 (3.15)
10	Q25	Congenital malformations of great arteries	6,150 (3.11)
**For Aalborg University Hospital**			
1	E10	Type 1 diabetes mellitus	11,245 (15.29)
2	G40	Epilepsy	7,716 (10.49)
3	C91	Lymphoid leukemia	7,492 (10.19)
4	Q21	Congenital malformations of cardiac septa	6,463 (8.79)
5	G80	Cerebral palsy	6,270 (8.53)
6	N18	Chronic kidney disease	2,555 (3.47)
7	Q24	Other congenital malformations of heart	2,268 (3.08)
8	K90	Intestinal malabsorption	2,124 (2.89)
9	K50	Crohn disease	2,032 (2.76)
10	Q87	Other specified congenital malformation syndromes affection multiple systems	1,865 (2.54)

Supplementary Table S22. Most common secondary diagnoses for outpatient visits with the four most common medical observation main diagnoses (ICD-10: Z01, Z03, Z13, and Z09). Most outpatient visits with these main diagnoses did not have secondary diagnoses. Percentages are relative to the number of outpatient visits with the main diagnosis. The percentages of outpatient visits that had a secondary diagnosis was 9,705 (2.7%) for main diagnosis Z01, 24,295 (8.6%) for main diagnosis Z03, 25,506 (16.1%) for main diagnosis Z13, and 24,304 (20.6%) for main diagnosis Z09.

	**ICD-10**		**n (%)**
**For main diagnosis Z01 (Other special examinations and investigations of persons without complaint)**			
1	Z13	Encounter for screening for other diseases and disorders	3,205 (0.89)
2	R47	Speech disturbances, not elsewhere classified	1,193 (0.33)
3	H65	Nonsuppurative otitis media	852 (0.24)
4	Z03	Encounter for medical observation for suspected diseases and conditions ruled out	541 (0.15)
5	H90	Conductive and sensorineural hearing loss	538 (0.15)
**For main diagnosis Z03 (Medical observation for suspected diseases/conditions, ruled out)**			
1	Z03	Encounter for medical observation for suspected diseases and conditions ruled out	1,422 (0.50)
2	R01	Cardiac murmurs and other cardiac sounds	1,362 (0.48)
3	R62	Lack of expected normal physiological development in childhood and adults	1,242 (0.44)
4	J45	Asthma	1,052 (0.37)
5	Z01	Encounter for other special examination without complaint, suspected or reported diagnosis	874 (0.31)
**For main diagnosis Z13 (Special screening examinations for other diseases and disorders)**			
1	Z00	Encounter for general examination without complaint, suspected or reported diagnosis	6,240 (3.93)
2	Z01	Encounter for other special examination without complaint, suspected or reported diagnosis	4,235 (2.67)
3	P92	Feeding problems of newborn	3,723 (2.35)
4	P59	Neonatal jaundice from other and unspecified causes	2,015 (1.27)
5	Z13	Encounter for screening for other diseases and disorders	1,831 (1.15)
**For main diagnosis Z09 (Follow-up examination after treatment for conditions other than malignant neoplasms)**			
1	Q21	Congenital malformations of cardiac septa	7,776 (6.45)
2	Q25	Congenital malformations of great arteries	4,066 (3.37)
3	Q20	Congenital malformations of cardiac chambers and connections	3,502 (2.90)
4	Q23	Congenital malformations of aortic and mitral valves	2,102 (1.74)
5	Q22	Congenital malformations of pulmonary and tricuspid valves	1,811 (1.50)

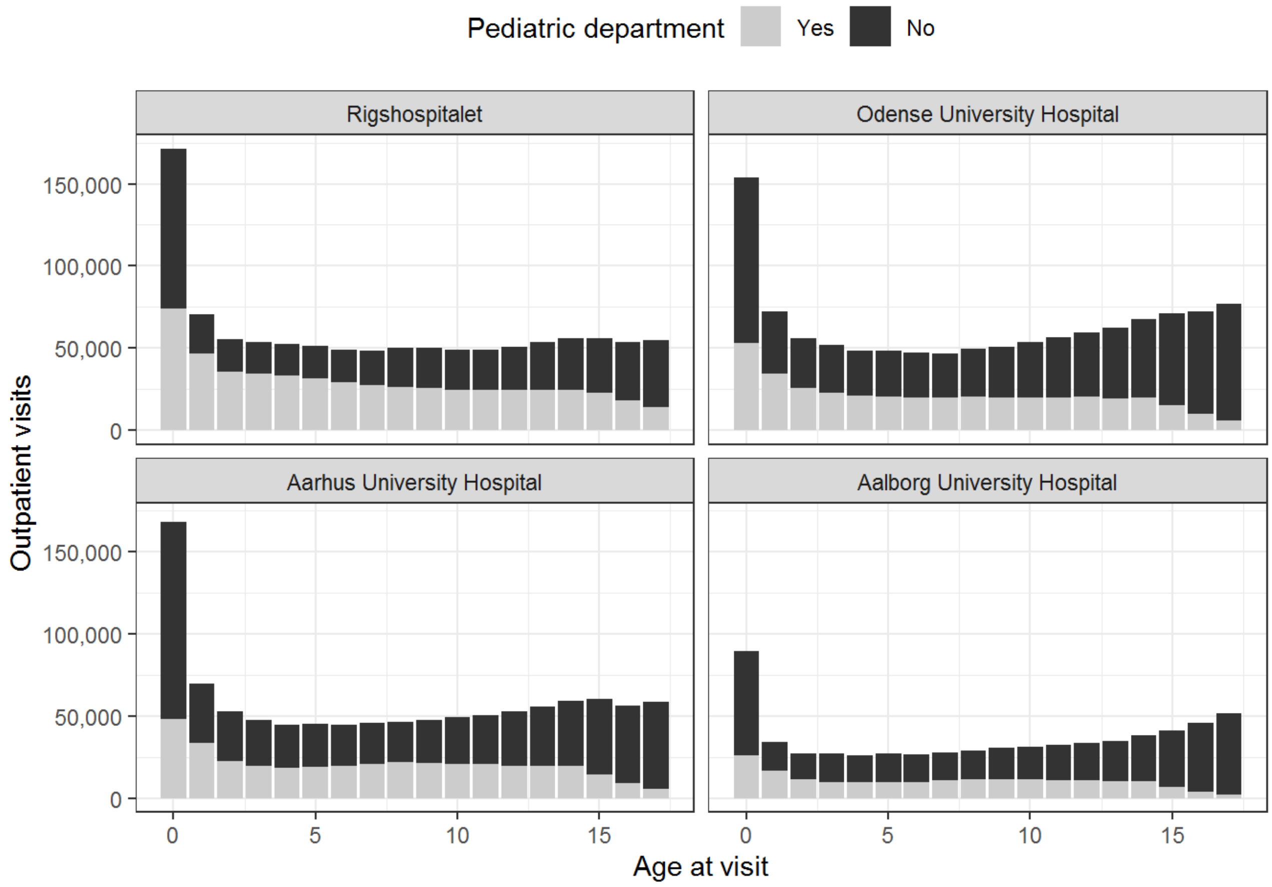

Supplementary Figure S29. Outpatient visits by age at time of visit grouped by whether the visit is at a pediatric department

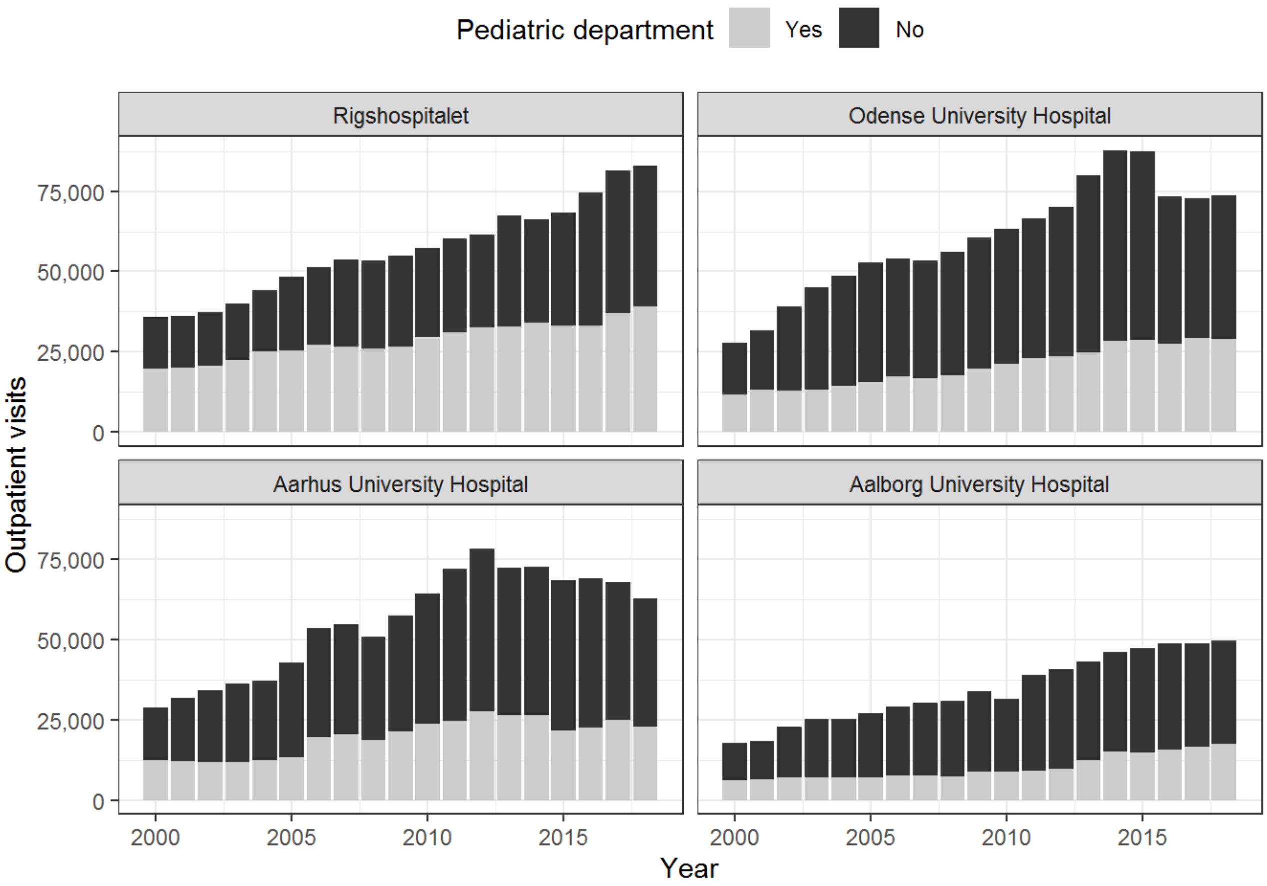

Supplementary Figure S30. Outpatient visits by year grouped by whether the visit is at a pediatric department

Supplementary Table S23. The patients' Region of residence at the start of the record for outpatient visits. The Region in which the hospital is placed is indicated in bold. Percentages are relative to the hospital

Region	Rigshospitalet	Odense University Hospital	Aarhus University Hospital	Aalborg University Hospital
Capital Region	**840,213 (78.1%)**	6,580 (0.6%)	3,511 (0.3%)	1,180 (0.2%)
Region Zealand	202,041 (18.8%)	13,069 (1.1%)	4,726 (0.4%)	568 (0.1%)
Region of Southern Denmark	16,593 (1.5%)	**1,093,154 (95.5%)**	71,573 (6.8%)	1,835 (0.3%)
Central Region Denmark	9,779 (0.9%)	23,150 (2.0%)	**926,175 (87.6%)**	6,832 (1.0%)
North Denmark Region	5,472 (0.5%)	6,264 (0.5%)	50,019 (4.7%)	**645,327 (98.4%)**
Missing	1,070 (0.1%)	2,323 (0.2%)	1,001 (0.1%)	289 (0.0%)

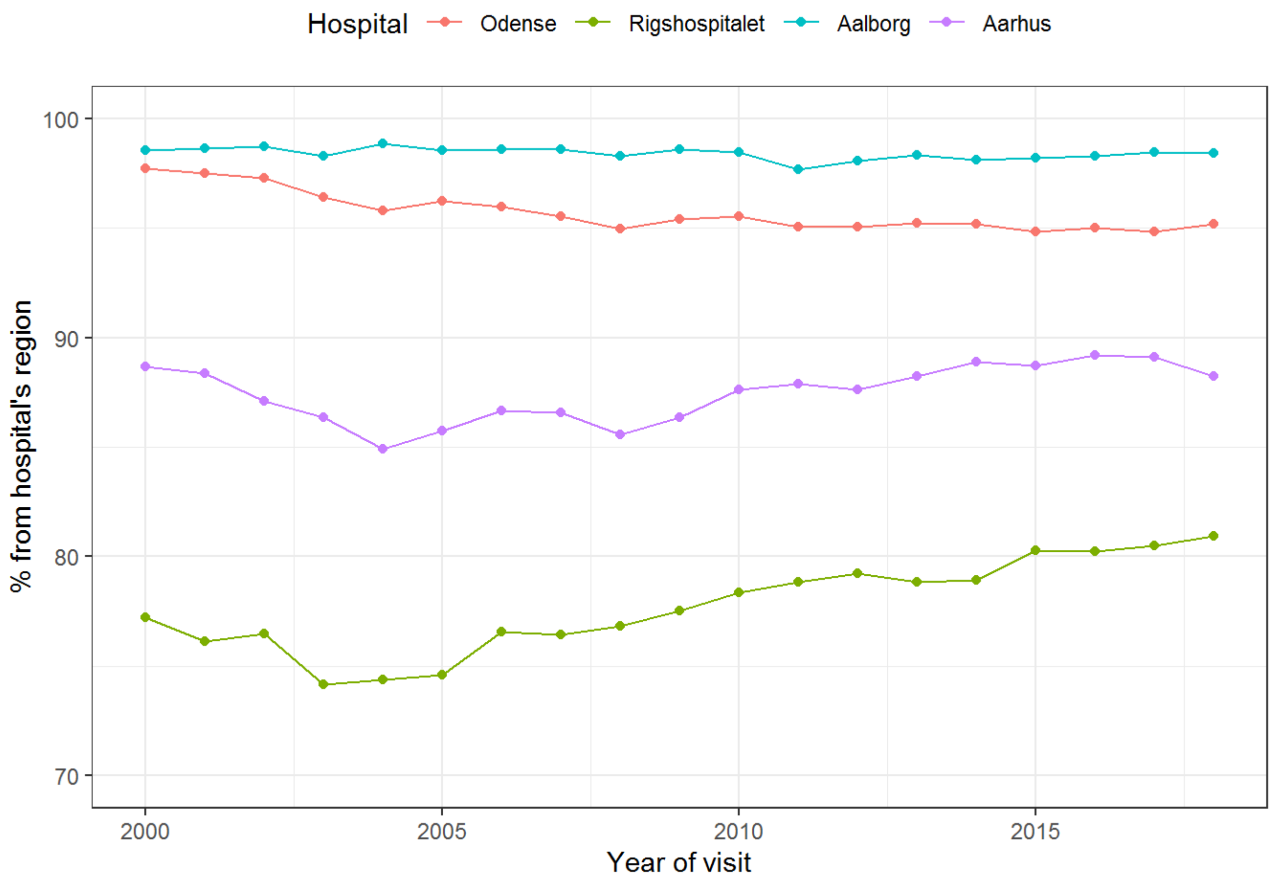

Supplementary Figure S31. Percent of outpatient visits starting in 2000-2018, where the patient lived in the hospital's region at the start of the corresponding record.

References

1. Kerbl, R. *et al.* Child Health Care Services in Austria. *Journal of Pediatrics* **177**, S35–S47 (2016).

2. Hejda, G. *et al.* Healthcare for children and adolescents in Poland. *Turkish Archives of Pediatrics* vol. 55 S63–S68 Preprint at https://doi.org/10.14744/TurkPediatriArs.2020.90692 (2020).

3. Pop, T. L. *et al.* Overview of the pediatric healthcare system in Romania. *Turkish Archives of Pediatrics* vol. 55 S69–S84 Preprint at https://doi.org/10.14744/TurkPediatriArs.2020.77775 (2020).

4. Zhang, Y. *et al.* Characteristics and workload of pediatricians in China. *Pediatrics* **144**, (2019).

5. Ehrich, J., Namazova-Baranova, L. & Pettoello-Mantovani, M. Introduction to "Diversity of Child Health Care in Europe: A Study of the European Paediatric Association/Union of National European Paediatric Societies and Associations". *Journal of Pediatrics* **177**, S1–S10 (2016).

6. Pettoello-Mantovani, M., Pop, T. L. & Hoey, H. Child healthcare in Europe, local diversities and collective potentials: A study of the European paediatric association/union of national European paediatric societies and associations. *Turkish Archives of Pediatrics* vol. 55 S1–S3 Preprint at https://doi.org/10.14744/TurkPediatriArs.2020.80106 (2020).

7. Efstathiou, E., Theophilou, L., Angeli, S. & Hadjipanayis, A. The child healthcare system in Cyprus. *Turkish Archives of Pediatrics* **55**, S24–S40 (2020).

8. Janda, J. Short Review of Child Health Care in Former Czechoslovakia and Czech Republic since 1993. *Journal of Pediatrics* **177**, S56–S59 (2016).

9. Mathiesen, P., Maarbjerg, S. F., Lykke, K. & Balslev, T. The Child Health System in Denmark: Current Problems and Successes. *Journal of Pediatrics* **177**, S60–S62 (2016).

10. Laszlo, S. *et al.* Child healthcare in Hungary. *Turkish Archives of Pediatrics* vol. 55 S41–S56 Preprint at https://doi.org/10.14744/TurkPediatriArs.2020.13333 (2020).

11. Staines, A. *et al.* Child Health Care in Ireland. *Journal of Pediatrics* **177**, S87–S106 (2016).

12. Corsello, G. *et al.* The Child Health Care System in Italy. *Journal of Pediatrics* **177**, S116–S126 (2016).

13. Juričič, M., Truden Dobrin, P., Paulin, S., Seher Zupančič, M. & Bratina, N. Health Care System for Children and Adolescents in Slovenia. *Journal of Pediatrics* **177**, S173–S186 (2016).

14. Jenni, O. G. & Sennhauser, F. H. Child Health Care in Switzerland. *Journal of Pediatrics* **177**, S203–S212 (2016).

15. Gualtieri, A., Antonelli, A. & Romeo, N. Healthcare programs in the eight European nations members of the world health organization small countries initiative for health: Andorra, Cyprus, Iceland, Luxembourg, Malta, Monaco, Montenegro, and San Marino. *Turkish Archives of Pediatrics* vol. 55 S85–S97 Preprint at https://doi.org/10.14744/TurkPediatriArs.2020.84829 (2020).

16. Norman, M. *et al.* Preterm birth in the Nordic countries—Capacity, management and outcome in neonatal care. *Acta Paediatrica, International Journal of Paediatrics* **112**, 1422–1433 (2023).

17. Kelly, L. E. *et al.* Perinatal health services organization for preterm births: A multinational comparison. *Journal of Perinatology* **37**, 762–768 (2017).

18. The Danish Health Data Authority. Sygehus-afdelingsklassifikation (SHAK). https://sundhedsdatastyrelsen.dk/da/rammer-og-retningslinjer/om-klassifikationer/afdelingsklassifikation.

19. Fløjstrup, M. *et al.* Increasing emergency hospital activity in Denmark, 2005-2016: A nationwide descriptive study. *BMJ Open* vol. 10 Preprint at https://doi.org/10.1136/bmjopen-2019-031409 (2020).

20. Rigsrevisionen. *Beretning Om Sundhedsplatformen* . https://www.ft.dk/statsrevisor/20171/beretning/sb17/bilag/1/1913849.pdf (2018).
